# Correction: Occurrence and distribution of anthropogenic persistent organic pollutants in coastal sediments and mud shrimps from the wetland of central Taiwan

**DOI:** 10.1371/journal.pone.0231279

**Published:** 2020-03-27

**Authors:** Shagnika Das, Andres H. Aria, Jing-O Cheng, Sami Souissi, Jiang-Shiou Hwang, Fung-Chi Ko

The second author's name is spelled incorrectly. The correct name is: Andres H. Aria. The correct citation is: Das S, Aria AH, Cheng J-O, Souissi S, Hwang J-S, Ko F-C (2020) Occurrence and distribution of anthropogenic persistent organic pollutants in coastal sediments and mud shrimps from the wetland of central Taiwan. PLoS ONE 15(1): e0227367. https://doi.org/10.1371/journal.pone.0227367
