# Correction: Correction: Occurrence and distribution of anthropogenic persistent organic pollutants in coastal sediments and mud shrimps from the wetland of central Taiwan

**DOI:** 10.1371/journal.pone.0233826

**Published:** 2020-05-21

**Authors:** The PLOS ONE Staff

The second author's name is spelled incorrectly in the correction notice. The publisher apologizes for the error. The correct name is: Andres H. Arias. The correct citation is: Das S, Arias AH, Cheng J-O, Souissi S, Hwang J-S, Ko F-C (2020) Occurrence and distribution of anthropogenic persistent organic pollutants in coastal sediments and mud shrimps from the wetland of central Taiwan. PLoS ONE 15(1): e0227367. https://doi.org/10.1371/journal.pone.0227367
