# Supplementary material for: Occurrence and distribution of anthropogenic persistent organic pollutants in coastal sediments and mud shrimps from the wetland of central Taiwan
Source: PLoS One. 2020 Jan 9;15(1):e0227367. doi: 10.1371/journal.pone.0227367 (PMC6956766; doi:10.1371/journal.pone.0227367)
Supplement: S1 Table — (DOCX) [file pone.0227367.s001.docx]

**Table S1. Sampling stations around the Changhua Industrial Park along the western coast of Taiwan with coordinates.**

| **Stations** | **Latitude (N^0^)** | **Longitude (E^0^)** | **Description** |
| --- | --- | --- | --- |
| **A** | 24.173 | 120.456 | Located in the northern periphery of the Industrial Park (the mud shrimp conservation area). |
| **B** | 24.164 | 120.458 | Similar location as station A, but outside the mud shrimp conservation area. |
| **C** | 24.124 | 120.418 | Located in the northern side within the Industrial Park. |
| **D** | 24.119 | 120.417 | Located in the southern side within the Industrial Park. |
| **E** | 24.015 | 120.349 | Located in the southern periphery of the Industrial Park |
