# Supplementary material for: Occurrence and distribution of anthropogenic persistent organic pollutants in coastal sediments and mud shrimps from the wetland of central Taiwan
Source: PLoS One. 2020 Jan 9;15(1):e0227367. doi: 10.1371/journal.pone.0227367 (PMC6956766; doi:10.1371/journal.pone.0227367)
Supplement: S2 Table — (DOCX) [file pone.0227367.s002.docx]

**Table S2.** Method detection limits (MDLs) and recovery (%) of PAHs analyzed in sediment samples in this study.

| No. | Compounds | Initial | MDL (ng) | Recovery (%) |
| --- | --- | --- | --- | --- |
| 1 | Naphthalene | Nap | 19.2 | 65.3±8.9 |
| 2 | 2-Methylnaphthalene | 2-MNap | 14.3 | 71.3±8.7 |
| 3 | 1-Methylnaphthalene | 1-MNap | 1.9 | 62.8±8.3 |
| 4 | 2,6-Dimethylnaphthalene | 2,6-MNap | 0.4 | 66.7±9.4 |
| 5 | 1,3-Dimethylnaphthalene | 1,3-MNap | 0.8 | 66.8±8.7 |
| 6 | 1,6-Dimethylnaphthalene | 1,6-MNap | 1.1 | 68.7±6.3 |
| 7 | 1,4-Dimethylnaphthalene | 1,4-MNap | 0.4 | 66.6±8.4 |
| 8 | 1,5-Dimethylnaphthalene | 1,5MNap | 0.4 | 67.5±8.9 |
| 9 | Acenaphthylene | Acy | 0.3 | 67.9±8.9 |
| 10 | 1,2-Dimethylnaphthalene | 1,2-MNap | 0.7 | 69.0±8.7 |
| 11 | Acenaphthene | Ace | 0.8 | 67.9±8.8 |
| 12 | Fluorene | Flu | 1.0 | 73.5±9.1 |
| 13 | 1-Methylfluorene | 1MFlu | 0.6 | 75.9±6.0 |
| 14 | Dibenzothiophene | DBT | 0.5 | 79.1±6.4 |
| 15 | Phenanthrene | Phe | 4.9 | 82.8±7.8 |
| 16 | Anthracene | Ant | 0.7 | 77.3±5.2 |
| 17 | 2-Methylphenanthrene | 2MPhe | 4.5 | 82.8±9.2 |
| 18 | 2-Methylanthracene | 2MAnt | 0.5 | 74.5±5.1 |
| 19 | 4,5-Methylenephenanthrene | 4,5-MPhe | 1.4 | 80.1±6.4 |
| 20 | 1-Methylanthracene | 1-MAnt | 0.9 | 70.7±4.2 |
| 21 | 1-Methylphenanthrene | 1MPhe | 1.0 | 82.6±6.0 |
| 22 | 4,6-Dimethyldibenzothiophene | 4,6-MDBT | 1.6 | 70.8±2.5 |
| 23 | Fluoranthene | Flo | 4.3 | 83.5±8.0 |
| 24 | Pyrene | Pyr | 2.5 | 83.6±9.7 |
| 25 | Retene | Ret | 1.9 | 82.7±8.0 |
| 26 | Benzo[a]fluorine | BaFu | 0.8 | 80.7±7.1 |
| 27 | Benzo[b]fluorine | BbFu | 0.9 | 73.7±7.4 |
| 28 | 1-Methylpyrene | 1MPye | 0.7 | 81.9±9.1 |
| 29 | Benz[a]anthracene | BaA | 0.6 | 83.1±9.7 |
| 30/31 | Chrysene+Triphenylene | Chr+TPh | 0.6 | 85.4±9.6 |
| 32/33 | 4/6-Methylchrysene | 4/6-NChr | 0.9 | 92.7±9.5 |
| 34 | Benzo[b]fluoranthene | BbF | 0.5 | 94.3±9.5 |

**Table S2.** (Continued) Method detection limits (MDLs) and recovery (%) of PAHs analyzed in sediment samples in this study.

| No. | Compounds | Initial | MDL (ng) | Recovery (%) |
| --- | --- | --- | --- | --- |
| 35 | Benzo[k]fluoranthene | BkF | 0.5 | 96.5±7.7 |
| 36 | Benzo[e]pyrene | BeP | 0.7 | 95.5±9.4 |
| 37 | Benzo[a]pyrene | BaP | 0.6 | 81.2±7.6 |
| 38 | Perylene | Per | 0.4 | 76.0±8.8 |
| 39 | Indeno[1,2,3-c,d]pyrene | IP | 0.5 | 77.4±7.9 |
| 40 | Dibenz[a,h]anthracene | DA | 0.4 | 84.5±9.9 |
| 41 | Benzo[g,h,i]perylene | BP | 0.4 | 84.7±10.5 |
| 42 | Coronene | Cor | 0.6 | 100.6±13.9 |
