# Supplementary material for: Occurrence and distribution of anthropogenic persistent organic pollutants in coastal sediments and mud shrimps from the wetland of central Taiwan
Source: PLoS One. 2020 Jan 9;15(1):e0227367. doi: 10.1371/journal.pone.0227367 (PMC6956766; doi:10.1371/journal.pone.0227367)
Supplement: S3 Table — (DOCX) [file pone.0227367.s003.docx]

**Table S3.** Method detection limits (MDLs) and recovery (%) of PCBs, OCPs and PBDEs analyzed in sediment samples in this study.

| Name | Cl-No. | MDL(pg) | Recovery% | Name | Cl-No. | MDL(pg) | Recovery% |
| --- | --- | --- | --- | --- | --- | --- | --- |
| #1 | Cl-1 | 6.8 | 60.1±12.4 | #70+76 | Cl-4 | 162.5 | 83.7±15.8 |
| #3 | Cl-1 | 7.4 | 63.9±11.7 | #74 | Cl-4 | 41.1 | 79.8±17.9 |
| #4+10 | Cl-2 | 2.0 | 63.7±11.0 | #77 | Cl-4 | 31.0 | 74.5±18.7 |
| #5+8 | Cl-2 | 18.5 | 71.9±10.6 | #82 | Cl-5 | 24.0 | 76.9±14.0 |
| #6 | Cl-2 | 4.0 | 68.8±10.3 | #84+92 | Cl-5 | 115.7 | 64.3±12.2 |
| #7+9 | Cl-2 | 2.5 | 68.7±11.9 | #85 | Cl-5 | 41.5 | 76.1±16.5 |
| #16+32 | Cl-3 | 14.2 | 77.3±11.3 | #87 | Cl-5 | 111.5 | 81.6±8.1 |
| #17 | Cl-3 | 13.6 | 73.9±11.9 | #89 | Cl-5 | 57.7 | 82.6±12.6 |
| #18 | Cl-3 | 8.4 | 74.4±11.4 | #91 | Cl-5 | 25.3 | 72.1±19.4 |
| #21+33 | Cl-3 | 9.2 | 80.9±11.9 | #95 | Cl-5 | 51.4 | 84.5±11.6 |
| #22 | Cl-3 | 16.1 | 83.1±14.4 | #97 | Cl-5 | 19.6 | 90.6±10.9 |
| #24 | Cl-3 | 3.9 | 70.5±9.6 | #99 | Cl-5 | 14.4 | 83.1±18.4 |
| #25 | Cl-3 | 9.8 | 75±16.1 | #101 | Cl-5 | 14.3 | 82.4±20.1 |
| #26 | Cl-3 | 8.3 | 71.8±13.5 | #110 | Cl-5 | 9.9 | 82.4±11.0 |
| #28 | Cl-3 | 9.2 | 76.4±16.2 | #118 | Cl-5 | 12.3 | 77.9±18.8 |
| #31 | Cl-3 | 10.2 | 75.7±14.8 | #132+153 | Cl-6 | 31.5 | 83.7±13.0 |
| #37 | Cl-3 | 9.9 | 87.4±12.2 | #136 | Cl-6 | 12.2 | 76.3±10.3 |
| #40 | Cl-4 | 22.9 | 84.2±15.6 | #138+163 | Cl-6 | 25.2 | 79.4±15.0 |
| #41+64+71 | Cl-4 | 101.8 | 86.8±8.3 | #141 | Cl-6 | 38.1 | 83.1±11.5 |
| #42* | Cl-4 | 15.7 | 82.9±13.5 | #149 | Cl-6 | 19.3 | 78.2±15.9 |
| #44 | Cl-4 | 17.3 | 84.7±9.5 | #151 | Cl-6 | 21.8 | 80.7±13.9 |
| #45 | Cl-4 | 14.9 | 79.1±11.5 | #170 | Cl-7 | 30.3 | 85.3±18.6 |
| #46 | Cl-4 | 21.0 | 75.2±12.9 | #171 | Cl-8 | 36.1 | 84±12.2 |
| #47 | Cl-4 | 152.7 | 82.8±18.6 | #174 | Cl-7 | 26.5 | 80.4±16.5 |
| #48 | Cl-4 | 15.3 | 80.2±10.0 | #176 | Cl-7 | 25.4 | 75.7±17.5 |
| #49 | Cl-4 | 11.7 | 77.6±14.6 | #177 | Cl-7 | 32.3 | 78.5±21.1 |
| #51 | Cl-4 | 37.7 | 79.3±9.4 | #178 | Cl-7 | 39.5 | 83±18.0 |
| #52 | Cl-4 | 12.1 | 78.1±13.3 | #180 | Cl-7 | 26.2 | 85.7±14.9 |
| #53 | Cl-4 | 29.7 | 78±12.4 | #182+187 | Cl-7 | 32.2 | 84.7±15.3 |
| #56+60 | Cl-4 | 18.4 | 89.7±13.3 | #183 | Cl-7 | 33.6 | 85.9±15.5 |
| #63 | Cl-4 | 16.8 | 87.4±13.7 | #185 | Cl-7 | 34.9 | 80.6±8.9 |
| #66 | Cl-4 | 11.5 | 82.8±14.5 | #190 | Cl-8 | 108.6 | 86±9.5 |

**Table S3.** (Continued) Method detection limits (MDLs) and recovery (%) of PCBs, OCPs and PBDEs analyzed in sediment samples in this study.

| Name | Cl-No. | MDL(pg) | Recovery% | Name | Br-No. | MDL(ng) | Recovery% |
| --- | --- | --- | --- | --- | --- | --- | --- |
| #193 | Cl-8 | 31.0 | 79.1±16.2 | BDE02 | Br-1 | 0.02 | 881.±23.4 |
| #194 | Cl-8 | 25.0 | 79.1±11.6 | BDE15 | Br-2 | 0.06 | 92.8±13.1 |
| #195 | Cl-8 | 50.1 | 78.7±20.1 | BDE17 | Br-3 | 0.46 | 106.5±9.2 |
| #196+203 | Cl-8 | 52.3 | 85.2±16.4 | BDE28 | Br-3 | 0.50 | 103.6±9.7 |
| #201 | Cl-8 | 47.9 | 87±16.9 | BDE47 | Br-4 | 0.26 | 107.0±7.4 |
| #202 | Cl-8 | 22.1 | 84.4±20.1 | BDE66 | Br-4 | 0.26 | 108.8±5.1 |
| #205 | Cl-8 | 42.4 | 87.3±3.6 | BDE71 | Br-4 | 0.35 | 105.1±9.7 |
| #206 | Cl-9 | 66.3 | 82.3±15.7 | BDE85 | Br-5 | 0.92 | 117.6±16.3 |
| Name | | MDL(ng) | Recovery% | BDE99 | Br-5 | 0.34 | 107.7±7.1 |
| Hexachlorobenzene (HCB) | | 0.5 | 72.3±11.2 | BDE100 | Br-5 | 0.54 | 105.5±10.7 |
| o,p'-DDE | | 0.1 | 86.3±11.6 | BDE138 | Br-6 | 0.81 | 106.7±27.6 |
| p,p'-DDE | | 0.1 | 85.6±10.1 | BDE153 | Br-6 | 0.53 | 104.0±17.5 |
| o,p'-DDD | | 0.1 | 86.1±10.1 | BDE154 | Br-6 | 0.63 | 98.9±11.4 |
| p,p'-DDD | | 0.1 | 83.4±12.0 | BDE183 | Br-7 | 0.47 | 105.9±27.6 |
| o,p'-DDT | | 0.1 | 73.4±12.9 | BDE190 | Br-7 | 0.39 | 88.0±24.0 |
| p,p'-DDT | | 0.1 | 73.5±12.8 | BDE203 | Br-8 | 0.27 | 86.3±33.3 |
|  | |  |  | BDE205 | Br-8 | 0.83 | 74.0±17.1 |
|  | |  |  | BDE206 | Br-9 | 1.0 | 67.3±0.7 |
|  | |  |  | BDE209 | Br-10 | 3.53 | 97.4±2.2 |
