# Supplementary material for: Occurrence and distribution of anthropogenic persistent organic pollutants in coastal sediments and mud shrimps from the wetland of central Taiwan
Source: PLoS One. 2020 Jan 9;15(1):e0227367. doi: 10.1371/journal.pone.0227367 (PMC6956766; doi:10.1371/journal.pone.0227367)
Supplement: S4 Table — (DOCX) [file pone.0227367.s004.docx]

**Table S4**. The quantitative ion and confirm ion in GC-MS/MS analysis for PCBs, OCPs and PBDEs in this study.

| PCBs | | | | | |
| --- | --- | --- | --- | --- | --- |
| Compounds | Quant. Ion (m/z) | Collision energy (eV) | Confirm ion (m/z) | Collision energy (eV) | C/Q ratio^b^ |
| Cl-1 | 188.0>152.0^a^ | 24 | 190.0>152.0 | 24 | 32.5% |
| Cl-2 | 222.0>152.0 | 24 | 224.0>152.0 | 24 | 63.7% |
| Cl-3 | 255.9>186.0 | 26 | 257.9>186.0 | 26 | 63.4% |
| Cl-4 | 289.9>219.9 | 26 | 291.9>221.9 | 26 | 64.3% |
| Cl-5 | 323.9>253.9 | 26 | 325.9>255.9 | 26 | 96.0% |
| Cl-6 | 359.9>289.9 | 28 | 361.9>291.9 | 28 | 48.3% |
| Cl-7 | 393.8>323.9 | 28 | 395.8>325.9 | 28 | 63.6% |
| Cl-8 | 427.8>357.8 | 28 | 429.8>359.8 | 28 | 79.5% |
| Cl-9 | 461.7>391.8 | 30 | 463.7>393.8 | 30 | 97.9% |
| Cl-10 | 497.7>427.8 | 30 | 463.7>429.8 | 30 | 63.7% |
| OCPs | | | | | |
| HCB | 283.8>248.8 | 24 | 283.8>213.8 | 28 | 70.2% |
| o,p’-DDE | 246.0>176.0 | 30 | 248.0>176.0 | 28 | 64.8% |
| p, p’-DDE | 246.0>176.0 | 30 | 248.0>176.0 | 28 | 48.3% |
| o,p’-DDD | 235.0>165.0 | 24 | 237.0>165.0 | 28 | 57.0% |
| p,p’-DDD | 235.0>165.0 | 24 | 237.0>165.0 | 28 | 62.1% |
| o,p’-DDT | 235.0>165.0 | 24 | 237.0>165.0 | 28 | 62.8% |
| p,p’-DDT | 235.0>165.0 | 24 | 237.0>165.0 | 28 | 60.3% |
| PBDEs | | | | | |
| Br-1 | 248.0>169.1 | 10 | 250.0>169.1 | 10 | 90.0% |
| Br-2 | 327.9>168.1 | 18 | 325.9>168.1 | 18 | 51.5% |
| Br-3 | 405.8>245.9 | 20 | 407.8>247.9 | 20 | 100.6% |
| Br-4 | 485.7>325.7 | 24 | 487.7>327.7 | 24 | 53.5% |
| Br-5 | 563.6>403.7 | 26 | 565.6>405.7 | 26 | 94.7% |
| Br-6 | 643.6>483.7 | 26 | 645.6>485.7 | 26 | 59.1% |
| Br-7 | 721.4>561.6 | 30 | 723.4>563.6 | 30 | 95.5% |
| Br-8 | 801.3>641.5 | 30 | 799.3>639.5 | 30 | 73.6% |
| Br-9 | 879.3>719.4 | 32 | 881.3>721.4 | 32 | 97.1% |
| Br-10 | 959.2>799.3 | 32 | 961.2>801.3 | 32 | 76.1% |
| MBDE209 | 971.2>811.3 | 32 | 973.2>813.3 | 32 | 71.2% |

^a^ precursor ion (m/z)> product ion (m/z)

^b^ signal intensity ratio of confirm and quantitative ion. In identification, default ion allowance variation was 30%.
