# Supplementary material for: Occurrence and distribution of anthropogenic persistent organic pollutants in coastal sediments and mud shrimps from the wetland of central Taiwan
Source: PLoS One. 2020 Jan 9;15(1):e0227367. doi: 10.1371/journal.pone.0227367 (PMC6956766; doi:10.1371/journal.pone.0227367)
Supplement: S1 Protocol — (DOCX) [file pone.0227367.s005.docx]

**Occurrence and distribution of anthropogenic persistent organic pollutants in coastal sediments and mud shrimps from the wetland of central Taiwan**

Shagnika Das^1,2^, Andres Aria^3, 4^, Jing-O Cheng^5^, Sami Souissi^2^, Jiang-Shiou Hwang^1, 6^, Fung Chi Ko^5, 7^*

^1^ Institute of Marine Biology, National Taiwan Ocean University, Keelung, Taiwan

^2^ University Lille, CNRS, University Littoral Cote d’Opale, UMR 8187, LOG, Laboratoire d’Océanologie et de Géosciences, 62930 Wimereux, France

^3^ [Argentine Institute of Oceanography](https://www.researchgate.net/institution/Argentine_Institute_of_Oceanography), Bahia Blanca, Argentina

^4^ National South University, Chemistry Dept., Area III, Bahía Blanca, Argentina

^5^ National Museum of Marine Biology and Aquarium, Checheng, Pingtung, Taiwan

^1^ Institute of Marine Biology, National Taiwan Ocean University, Keelung, Taiwan

^6^ Center of Excellence for the Oceans, National Taiwan Ocean University,
Keelung 20224, Taiwan

^7^ Institute of Marine Biology, National Dong-Hwa University, Pingtung, Taiwan

1. **Sampling station description**

**Table S1** Sampling stations around the Changhua Industrial Park along the western coast of Taiwan with coordinates.

| **Stations** | **Latitude (N^0^)** | **Longitude (E^0^)** | **Description** |
| --- | --- | --- | --- |
| **A** | 24.173 | 120.456 | Located in the northern periphery of the Industrial Park (the mud shrimp conservation area). |
| **B** | 24.164 | 120.458 | Similar location as station A, but outside the mud shrimp conservation area. |
| **C** | 24.124 | 120.418 | Located in the northern side within the Industrial Park. |
| **D** | 24.119 | 120.417 | Located in the southern side within the Industrial Park. |
| **E** | 24.015 | 120.349 | Located in the southern periphery of the Industrial Park |

1. **Instrument analytical procedure**

After the extraction and purification, PAHs, PCBs, OCPs, and PBDEs in sediment and shrimp samples were analyzed using analytical methods from previous studies with some modification [1, 2, 3]. Details are as follows:

- 1. **PAHs**

PAHs were analyzed using a Varian CP3800-320MS with EI source and a VF-5ms column (30m x 0.25 mm I.D.; 0.25μm film thickness). The oven temperature was programmed to an initial temperature of 50^o^C, increased to 120^o^C at 5^o^C min^-1^ , increased to 280^o^C at 10^o^C min^-1^ , and finally increased to 310^o^C at 5^o^C min^-1^ and held for 9 min. Helium was used as the carrier gas, with a 1 ml min^-1^ flow rate. The temperatures of the injector, transfer line, and source were all 250^o^C.

- 1. **PCBs**

PCBs were analyzed using a Shimadzu TQ8050 with EI source and a DB-5 column (60 m x 0.25 mm I.D.; 0.25 μm film thickness). The oven temperature was programmed to an initial temperature of 110^o^C, held for 2 min, , increased at a rate of 20^o^C min^-1^ to 170^o^C, held for one min, , increased at a rate of 1.5^o^C min^-1^ to 225^o^C. held for one min, increased at a rate of 2^o^C min^-1^ to 235^o^C, held for one min, and finally increased at a rate of 5^o^C min^-1^ to 310^o^C and held for 5.33 min. Helium was used as the carrier gas, with a 1 ml min^-1^ flow rate. The temperature of the injector, transfer line, and source were 270^o^C, 290^o^C, and 230^o^C, respectively. The quantitative ion and confirm ion in GC-MS/MS analysis are shown in Table S4.

- 1. **OCPs**

OCPs were analyzed using a Shimadzu TQ8050 with EI source and a VF-5ms column (30 m x 0.25 mm I.D.; 0.25 μm film thickness). The oven temperature was programmed to an initial temperature of 50^o^C, held for 1 min, increased at a rate of 25^o^C min^-1^ to 125^o^C, and finally increased at a rate of 10^o^C min^-1^ to 310^o^C and held for 2 min. Helium was used as the carrier gas, with a 1 ml min^-1^ flow rate. The temperature of the injector, transfer line, and source were 250^o^C, 250^o^C, and 230^o^C, respectively. The quantitative ion and confirm ion in GC-MS/MS analysis are shown in Table S4.

- 1. **PBDEs**

OCPs were analyzed using a Shimadzu TQ8050 with EI source and a VF-5ht column (15m x 0.25 mm I.D.; 0.1μm film thickness). The oven temperature was programmed to an initial temperature of 40^o^C, held for 1.5min, increased at a rate of 45^o^C min^-1^ to 140^o^C, and finally increased at a rate of 15^o^C min^-1^ to 330^o^C and held for 4 min. Helium was used as the carrier gas, with a 2 ml min^-1^ flow rate. The temperature of the injector, transfer line, and source were all 300^o^C. The quantitative ion and confirm ion in GC-MS/MS analysis are shown in Table S4.

**Table S2.** Method detection limits (MDLs) and recovery (%) of PAHs analyzed in sediment samples in this study.

| No. | Compounds | Initial | MDL (ng) | Recovery (%) |
| --- | --- | --- | --- | --- |
| 1 | Naphthalene | Nap | 19.2 | 65.3±8.9 |
| 2 | 2-Methylnaphthalene | 2-MNap | 14.3 | 71.3±8.7 |
| 3 | 1-Methylnaphthalene | 1-MNap | 1.9 | 62.8±8.3 |
| 4 | 2,6-Dimethylnaphthalene | 2,6-MNap | 0.4 | 66.7±9.4 |
| 5 | 1,3-Dimethylnaphthalene | 1,3-MNap | 0.8 | 66.8±8.7 |
| 6 | 1,6-Dimethylnaphthalene | 1,6-MNap | 1.1 | 68.7±6.3 |
| 7 | 1,4-Dimethylnaphthalene | 1,4-MNap | 0.4 | 66.6±8.4 |
| 8 | 1,5-Dimethylnaphthalene | 1,5MNap | 0.4 | 67.5±8.9 |
| 9 | Acenaphthylene | Acy | 0.3 | 67.9±8.9 |
| 10 | 1,2-Dimethylnaphthalene | 1,2-MNap | 0.7 | 69.0±8.7 |
| 11 | Acenaphthene | Ace | 0.8 | 67.9±8.8 |
| 12 | Fluorene | Flu | 1.0 | 73.5±9.1 |
| 13 | 1-Methylfluorene | 1MFlu | 0.6 | 75.9±6.0 |
| 14 | Dibenzothiophene | DBT | 0.5 | 79.1±6.4 |
| 15 | Phenanthrene | Phe | 4.9 | 82.8±7.8 |
| 16 | Anthracene | Ant | 0.7 | 77.3±5.2 |
| 17 | 2-Methylphenanthrene | 2MPhe | 4.5 | 82.8±9.2 |
| 18 | 2-Methylanthracene | 2MAnt | 0.5 | 74.5±5.1 |
| 19 | 4,5-Methylenephenanthrene | 4,5-MPhe | 1.4 | 80.1±6.4 |
| 20 | 1-Methylanthracene | 1-MAnt | 0.9 | 70.7±4.2 |
| 21 | 1-Methylphenanthrene | 1MPhe | 1.0 | 82.6±6.0 |
| 22 | 4,6-Dimethyldibenzothiophene | 4,6-MDBT | 1.6 | 70.8±2.5 |
| 23 | Fluoranthene | Flo | 4.3 | 83.5±8.0 |
| 24 | Pyrene | Pyr | 2.5 | 83.6±9.7 |
| 25 | Retene | Ret | 1.9 | 82.7±8.0 |
| 26 | Benzo[a]fluorine | BaFu | 0.8 | 80.7±7.1 |
| 27 | Benzo[b]fluorine | BbFu | 0.9 | 73.7±7.4 |
| 28 | 1-Methylpyrene | 1MPye | 0.7 | 81.9±9.1 |
| 29 | Benz[a]anthracene | BaA | 0.6 | 83.1±9.7 |
| 30/31 | Chrysene+Triphenylene | Chr+TPh | 0.6 | 85.4±9.6 |
| 32/33 | 4/6-Methylchrysene | 4/6-NChr | 0.9 | 92.7±9.5 |
| 34 | Benzo[b]fluoranthene | BbF | 0.5 | 94.3±9.5 |

**Table S2.** (Continued) Method detection limits (MDLs) and recovery (%) of PAHs analyzed in sediment samples in this study.

| No. | Compounds | Initial | MDL (ng) | Recovery (%) |
| --- | --- | --- | --- | --- |
| 35 | Benzo[k]fluoranthene | BkF | 0.5 | 96.5±7.7 |
| 36 | Benzo[e]pyrene | BeP | 0.7 | 95.5±9.4 |
| 37 | Benzo[a]pyrene | BaP | 0.6 | 81.2±7.6 |
| 38 | Perylene | Per | 0.4 | 76.0±8.8 |
| 39 | Indeno[1,2,3-c,d]pyrene | IP | 0.5 | 77.4±7.9 |
| 40 | Dibenz[a,h]anthracene | DA | 0.4 | 84.5±9.9 |
| 41 | Benzo[g,h,i]perylene | BP | 0.4 | 84.7±10.5 |
| 42 | Coronene | Cor | 0.6 | 100.6±13.9 |

**Table S3.** Method detection limits (MDLs) and recovery (%) of PCBs, OCPs and PBDEs analyzed in sediment samples in this study.

| Name | Cl-No. | MDL(pg) | Recovery% | Name | Cl-No. | MDL(pg) | Recovery% |
| --- | --- | --- | --- | --- | --- | --- | --- |
| #1 | Cl-1 | 6.8 | 60.1±12.4 | #70+76 | Cl-4 | 162.5 | 83.7±15.8 |
| #3 | Cl-1 | 7.4 | 63.9±11.7 | #74 | Cl-4 | 41.1 | 79.8±17.9 |
| #4+10 | Cl-2 | 2.0 | 63.7±11.0 | #77 | Cl-4 | 31.0 | 74.5±18.7 |
| #5+8 | Cl-2 | 18.5 | 71.9±10.6 | #82 | Cl-5 | 24.0 | 76.9±14.0 |
| #6 | Cl-2 | 4.0 | 68.8±10.3 | #84+92 | Cl-5 | 115.7 | 64.3±12.2 |
| #7+9 | Cl-2 | 2.5 | 68.7±11.9 | #85 | Cl-5 | 41.5 | 76.1±16.5 |
| #16+32 | Cl-3 | 14.2 | 77.3±11.3 | #87 | Cl-5 | 111.5 | 81.6±8.1 |
| #17 | Cl-3 | 13.6 | 73.9±11.9 | #89 | Cl-5 | 57.7 | 82.6±12.6 |
| #18 | Cl-3 | 8.4 | 74.4±11.4 | #91 | Cl-5 | 25.3 | 72.1±19.4 |
| #21+33 | Cl-3 | 9.2 | 80.9±11.9 | #95 | Cl-5 | 51.4 | 84.5±11.6 |
| #22 | Cl-3 | 16.1 | 83.1±14.4 | #97 | Cl-5 | 19.6 | 90.6±10.9 |
| #24 | Cl-3 | 3.9 | 70.5±9.6 | #99 | Cl-5 | 14.4 | 83.1±18.4 |
| #25 | Cl-3 | 9.8 | 75±16.1 | #101 | Cl-5 | 14.3 | 82.4±20.1 |
| #26 | Cl-3 | 8.3 | 71.8±13.5 | #110 | Cl-5 | 9.9 | 82.4±11.0 |
| #28 | Cl-3 | 9.2 | 76.4±16.2 | #118 | Cl-5 | 12.3 | 77.9±18.8 |
| #31 | Cl-3 | 10.2 | 75.7±14.8 | #132+153 | Cl-6 | 31.5 | 83.7±13.0 |
| #37 | Cl-3 | 9.9 | 87.4±12.2 | #136 | Cl-6 | 12.2 | 76.3±10.3 |
| #40 | Cl-4 | 22.9 | 84.2±15.6 | #138+163 | Cl-6 | 25.2 | 79.4±15.0 |
| #41+64+71 | Cl-4 | 101.8 | 86.8±8.3 | #141 | Cl-6 | 38.1 | 83.1±11.5 |
| #42* | Cl-4 | 15.7 | 82.9±13.5 | #149 | Cl-6 | 19.3 | 78.2±15.9 |
| #44 | Cl-4 | 17.3 | 84.7±9.5 | #151 | Cl-6 | 21.8 | 80.7±13.9 |
| #45 | Cl-4 | 14.9 | 79.1±11.5 | #170 | Cl-7 | 30.3 | 85.3±18.6 |
| #46 | Cl-4 | 21.0 | 75.2±12.9 | #171 | Cl-8 | 36.1 | 84±12.2 |
| #47 | Cl-4 | 152.7 | 82.8±18.6 | #174 | Cl-7 | 26.5 | 80.4±16.5 |
| #48 | Cl-4 | 15.3 | 80.2±10.0 | #176 | Cl-7 | 25.4 | 75.7±17.5 |
| #49 | Cl-4 | 11.7 | 77.6±14.6 | #177 | Cl-7 | 32.3 | 78.5±21.1 |
| #51 | Cl-4 | 37.7 | 79.3±9.4 | #178 | Cl-7 | 39.5 | 83±18.0 |
| #52 | Cl-4 | 12.1 | 78.1±13.3 | #180 | Cl-7 | 26.2 | 85.7±14.9 |
| #53 | Cl-4 | 29.7 | 78±12.4 | #182+187 | Cl-7 | 32.2 | 84.7±15.3 |
| #56+60 | Cl-4 | 18.4 | 89.7±13.3 | #183 | Cl-7 | 33.6 | 85.9±15.5 |
| #63 | Cl-4 | 16.8 | 87.4±13.7 | #185 | Cl-7 | 34.9 | 80.6±8.9 |
| #66 | Cl-4 | 11.5 | 82.8±14.5 | #190 | Cl-8 | 108.6 | 86±9.5 |

**Table S3.** (Continued) Method detection limits (MDLs) and recovery (%) of PCBs, OCPs and PBDEs analyzed in sediment samples in this study.

| Name | Cl-No. | MDL(pg) | Recovery% | Name | Br-No. | MDL(ng) | Recovery% |
| --- | --- | --- | --- | --- | --- | --- | --- |
| #193 | Cl-8 | 31.0 | 79.1±16.2 | BDE02 | Br-1 | 0.02 | 881.±23.4 |
| #194 | Cl-8 | 25.0 | 79.1±11.6 | BDE15 | Br-2 | 0.06 | 92.8±13.1 |
| #195 | Cl-8 | 50.1 | 78.7±20.1 | BDE17 | Br-3 | 0.46 | 106.5±9.2 |
| #196+203 | Cl-8 | 52.3 | 85.2±16.4 | BDE28 | Br-3 | 0.50 | 103.6±9.7 |
| #201 | Cl-8 | 47.9 | 87±16.9 | BDE47 | Br-4 | 0.26 | 107.0±7.4 |
| #202 | Cl-8 | 22.1 | 84.4±20.1 | BDE66 | Br-4 | 0.26 | 108.8±5.1 |
| #205 | Cl-8 | 42.4 | 87.3±3.6 | BDE71 | Br-4 | 0.35 | 105.1±9.7 |
| #206 | Cl-9 | 66.3 | 82.3±15.7 | BDE85 | Br-5 | 0.92 | 117.6±16.3 |
| Name | | MDL(ng) | Recovery% | BDE99 | Br-5 | 0.34 | 107.7±7.1 |
| Hexachlorobenzene (HCB) | | 0.5 | 72.3±11.2 | BDE100 | Br-5 | 0.54 | 105.5±10.7 |
| o,p'-DDE | | 0.1 | 86.3±11.6 | BDE138 | Br-6 | 0.81 | 106.7±27.6 |
| p,p'-DDE | | 0.1 | 85.6±10.1 | BDE153 | Br-6 | 0.53 | 104.0±17.5 |
| o,p'-DDD | | 0.1 | 86.1±10.1 | BDE154 | Br-6 | 0.63 | 98.9±11.4 |
| p,p'-DDD | | 0.1 | 83.4±12.0 | BDE183 | Br-7 | 0.47 | 105.9±27.6 |
| o,p'-DDT | | 0.1 | 73.4±12.9 | BDE190 | Br-7 | 0.39 | 88.0±24.0 |
| p,p'-DDT | | 0.1 | 73.5±12.8 | BDE203 | Br-8 | 0.27 | 86.3±33.3 |
|  | |  |  | BDE205 | Br-8 | 0.83 | 74.0±17.1 |
|  | |  |  | BDE206 | Br-9 | 1.0 | 67.3±0.7 |
|  | |  |  | BDE209 | Br-10 | 3.53 | 97.4±2.2 |

**Table S4**. The quantitative ion and confirm ion in GC-MS/MS analysis for PCBs, OCPs and PBDEs in this study.

| PCBs | | | | | |
| --- | --- | --- | --- | --- | --- |
| Compounds | Quant. Ion (m/z) | Collision energy (eV) | Confirm ion (m/z) | Collision energy (eV) | C/Q ratio^b^ |
| Cl-1 | 188.0>152.0^a^ | 24 | 190.0>152.0 | 24 | 32.5% |
| Cl-2 | 222.0>152.0 | 24 | 224.0>152.0 | 24 | 63.7% |
| Cl-3 | 255.9>186.0 | 26 | 257.9>186.0 | 26 | 63.4% |
| Cl-4 | 289.9>219.9 | 26 | 291.9>221.9 | 26 | 64.3% |
| Cl-5 | 323.9>253.9 | 26 | 325.9>255.9 | 26 | 96.0% |
| Cl-6 | 359.9>289.9 | 28 | 361.9>291.9 | 28 | 48.3% |
| Cl-7 | 393.8>323.9 | 28 | 395.8>325.9 | 28 | 63.6% |
| Cl-8 | 427.8>357.8 | 28 | 429.8>359.8 | 28 | 79.5% |
| Cl-9 | 461.7>391.8 | 30 | 463.7>393.8 | 30 | 97.9% |
| Cl-10 | 497.7>427.8 | 30 | 463.7>429.8 | 30 | 63.7% |
| OCPs | | | | | |
| HCB | 283.8>248.8 | 24 | 283.8>213.8 | 28 | 70.2% |
| o,p’-DDE | 246.0>176.0 | 30 | 248.0>176.0 | 28 | 64.8% |
| p, p’-DDE | 246.0>176.0 | 30 | 248.0>176.0 | 28 | 48.3% |
| o,p’-DDD | 235.0>165.0 | 24 | 237.0>165.0 | 28 | 57.0% |
| p,p’-DDD | 235.0>165.0 | 24 | 237.0>165.0 | 28 | 62.1% |
| o,p’-DDT | 235.0>165.0 | 24 | 237.0>165.0 | 28 | 62.8% |
| p,p’-DDT | 235.0>165.0 | 24 | 237.0>165.0 | 28 | 60.3% |
| PBDEs | | | | | |
| Br-1 | 248.0>169.1 | 10 | 250.0>169.1 | 10 | 90.0% |
| Br-2 | 327.9>168.1 | 18 | 325.9>168.1 | 18 | 51.5% |
| Br-3 | 405.8>245.9 | 20 | 407.8>247.9 | 20 | 100.6% |
| Br-4 | 485.7>325.7 | 24 | 487.7>327.7 | 24 | 53.5% |
| Br-5 | 563.6>403.7 | 26 | 565.6>405.7 | 26 | 94.7% |
| Br-6 | 643.6>483.7 | 26 | 645.6>485.7 | 26 | 59.1% |
| Br-7 | 721.4>561.6 | 30 | 723.4>563.6 | 30 | 95.5% |
| Br-8 | 801.3>641.5 | 30 | 799.3>639.5 | 30 | 73.6% |
| Br-9 | 879.3>719.4 | 32 | 881.3>721.4 | 32 | 97.1% |
| Br-10 | 959.2>799.3 | 32 | 961.2>801.3 | 32 | 76.1% |
| MBDE209 | 971.2>811.3 | 32 | 973.2>813.3 | 32 | 71.2% |

^a^ precursor ion (m/z)> product ion (m/z)

^b^ signal intensity ratio of confirm and quantitative ion. In identification, default ion allowance variation was 30%.

**Reference**

1. Cheng, J.-O., Ko, F.-C., Lee, C.-L., Fang, M.-D., 2016. Atmospheric polycyclic aromatic hydrocarbons (PAHs) of southern Taiwan in relation to monsoons. Environmental Science and Pollution Research 23, 15675-15688.

2. He, W., Bai, Z.-L., Liu, W.-X., Kong, X.-Z., Yang, B., Yang, C., Jørgensen, S.E., Xu, F.-L., 2016. Occurrence, spatial distribution, sources, and risks of polychlorinated biphenyls and heavy metals in surface sediments from a large eutrophic Chinese lake (Lake Chaohu). Environmental Science and Pollution Research 23, 10335-10348.

3. Wang, D., Miao, X., Li, Q.X., 2008. Analysis of organochlorine pesticides in coral (Porites evermanni) samples using accelerated solvent extraction and gas chromatography/ion trap mass spectrometry. Archives of Environmental Contamination and Toxicology 54, 211-218.
