# Supplementary material for: Occurrence and distribution of anthropogenic persistent organic pollutants in coastal sediments and mud shrimps from the wetland of central Taiwan
Source: PLoS One. 2020 Jan 9;15(1):e0227367. doi: 10.1371/journal.pone.0227367 (PMC6956766; doi:10.1371/journal.pone.0227367)
Supplement: Attachment — Submitted filename: PONE-D-19-27465_reviewer.pdf [file pone.0227367.s006.pdf]

PLOS ONE

Occurrence and distribution of anthropogenic persistent organic pollutants in coastal sediments and mud shrimps from the wetland of central Taiwan

--Manuscript Draft--

**Manuscript Number:**	PONE-D-19-27465
**Article Type:**	Research Article
**Full Title:**	Occurrence and distribution of anthropogenic persistent organic pollutants in coastal sediments and mud shrimps from the wetland of central Taiwan
**Short Title:**	POP distribution in coastal sediment and benthos: potential impact of industrial to wetland ecosystem
**Corresponding Author:**	Fung-Chi Ko, PhD<br>National Dong Hwa University<br>Pingtung, TAIWAN
**Keywords:**	POPs; PAHs; PBDEs; PCBs; sediment; mud shrimp
**Abstract:**	Sediment profile and mud shrimp (Austinogebia edulis) from the coastal wetland of central Taiwan in 2017 and 2018 were analyzed for concentration, source, and composition of persistent organic pollutants (POPs) including polycyclic aromatic and hydrocarbon (PAHs), polybrominated diphenyl ethers (PBDEs), organochlorine pesticides (DDT and HCB), and polychlorobiphenyls (PCBs). Results suggest that the determining factor for spatial distribution of POPs might be proximity to contaminant sources near industrial zones. Based on molecular indices, PAHs were substantially of both pyrolytic and petrogenic origins. The main sources for PCBs were Aroclor 1016 and 1260 and the congener BDE 209 was the dominant component among PBDE congeners. The risk assessment on mud shrimp illustrated a borderline threat, with DDT concentrations almost reaching standardized values of Effects Range-Low (ERL). Bioaccumulation factors for DDTs and PCBs (17.33 and 54.59, respectively) were higher than other POPs in this study. Further study is essential to assess and understand the impact of these chemicals on the wetland ecosystem near this heavily industrialized area.
**Order of Authors:**	Shagnika Das
	Andres Aria
	Jing-O Cheng
	Sami Souissi
	Jiang-Shiou Hwang
	Fung-Chi Ko, PhD
**Additional Information:**	
**Question**	**Response**
**Financial Disclosure**<br><br>Enter a financial disclosure statement that describes the sources of funding for the work included in this submission. Review the submission guidelines for detailed requirements. View published research articles from *PLOS ONE* for specific examples.<br><br>This statement is required for submission and **will appear in the published article** if	The funders had no role in study design, data collection and analysis, decision to publish, or preparation of the manuscript.

Powered by Editorial Manager® and ProduXion Manager® from Aries Systems Corporation

the submission is accepted. Please make sure it is accurate.<br><br>**Unfunded studies**<br>Enter: *The author(s) received no specific funding for this work.*<br><br>**Funded studies**<br>Enter a statement with the following details:<br>• Initials of the authors who received each award<br>• Grant numbers awarded to each author<br>• The full name of each funder<br>• URL of each funder website<br>• Did the sponsors or funders play any role in the study design, data collection and analysis, decision to publish, or preparation of the manuscript?<br>• **NO** - Include this sentence at the end of your statement: *The funders had no role in study design, data collection and analysis, decision to publish, or preparation of the manuscript.*<br>• **YES** - Specify the role(s) played.<br><br>* typeset	
**Competing Interests**<br><br>Use the instructions below to enter a competing interest statement for this submission. On behalf of all authors, disclose any competing interests that could be perceived to bias this work—acknowledging all financial support and any other relevant financial or non-financial competing interests.<br><br>This statement **will appear in the published article** if the submission is accepted. Please make sure it is accurate. View published research articles from *PLOS ONE* for specific examples.	The authors have declared that no competing interests exist.

Powered by Editorial Manager® and ProduXion Manager® from Aries Systems Corporation

**NO authors have competing interests**<br><br>Enter: *The authors have declared that no competing interests exist.*<br><br>**Authors with competing interests**<br><br>Enter competing interest details beginning with this statement:<br><br>*I have read the journal's policy and the authors of this manuscript have the following competing interests: [insert competing interests here]*<br><br>* typeset	
**Ethics Statement**<br><br>Enter an ethics statement for this submission. This statement is required if the study involved:<br><br>• Human participants<br>• Human specimens or tissue<br>• Vertebrate animals or cephalopods<br>• Vertebrate embryos or tissues<br>• Field research<br><br>Write "N/A" if the submission does not require an ethics statement.<br><br>General guidance is provided below. Consult the submission guidelines for detailed instructions. **Make sure that all information entered here is included in the Methods section of the manuscript.**	N/A

**Format for specific study types**<br><br>**Human Subject Research (involving human participants and/or tissue)**<br>• Give the name of the institutional review board or ethics committee that approved the study<br>• Include the approval number and/or a statement indicating approval of this research<br>• Indicate the form of consent obtained (written/oral) or the reason that consent was not obtained (e.g. the data were analyzed anonymously)<br><br>**Animal Research (involving vertebrate animals, embryos or tissues)**<br>• Provide the name of the Institutional Animal Care and Use Committee (IACUC) or other relevant ethics board that reviewed the study protocol, and indicate whether they approved this research or granted a formal waiver of ethical approval<br>• Include an approval number if one was obtained<br>• If the study involved *non-human primates*, add *additional details* about animal welfare and steps taken to ameliorate suffering<br>• If anesthesia, euthanasia, or any kind of animal sacrifice is part of the study, include briefly which substances and/or methods were applied<br><br>**Field Research**<br><br>Include the following details if this study involves the collection of plant, animal, or other materials from a natural setting:<br>• Field permit number<br>• Name of the institution or relevant body that granted permission	
**Data Availability**<br><br>Authors are required to make all data underlying the findings described fully available, without restriction, and from the time of publication. PLOS allows rare exceptions to address legal and ethical concerns. See the PLOS Data Policy and FAQ for detailed information.	Yes - all data are fully available without restriction

A Data Availability Statement describing where the data can be found is required at submission. Your answers to this question constitute the Data Availability Statement and **will be published in the article**, if accepted.<br><br>**Important:** Stating 'data available on request from the author' is not sufficient. If your data are only available upon request, select 'No' for the first question and explain your exceptional situation in the text box.<br><br>Do the authors confirm that all data underlying the findings described in their manuscript are fully available without restriction?	
**Describe where the data may be found in full sentences. If you are copying our sample text, replace any instances of XXX with the appropriate details.**<br><br>• If the data are **held or will be held in a public repository**, include URLs, accession numbers or DOIs. If this information will only be available after acceptance, indicate this by ticking the box below. For example: *All XXX files are available from the XXX database (accession number(s) XXX, XXX.).*<br>• If the data are all contained **within the manuscript and/or Supporting Information files**, enter the following: *All relevant data are within the manuscript and its Supporting Information files.*<br>• If neither of these applies but you are able to provide **details of access elsewhere**, with or without limitations, please do so. For example:<br><br>*Data cannot be shared publicly because of [XXX]. Data are available from the XXX Institutional Data Access / Ethics Committee (contact via XXX) for researchers who meet the criteria for access to confidential data.*<br><br>*The data underlying the results presented in the study are available from (include the name of the third party*	All relevant data are within the manuscript and its Supporting Information files.

and contact information or URL).<br>• This text is appropriate if the data are owned by a third party and authors do not have permission to share the data.<br><br>* typeset	
Additional data availability information:	Tick here if the URLs/accession numbers/DOIs will be available only after acceptance of the manuscript for publication so that we can ensure their inclusion before publication.

Powered by Editorial Manager® and ProduXion Manager® from Aries Systems Corporation

Manuscript Click here to download Manuscript final text.docx

Occurrence and distribution of anthropogenic persistent organic pollutants in coastal sediments and mud shrimps from the wetland of central Taiwan

Shagnika Das[1,2], Andres Aria[3, 4], Jing-O Cheng[5], Sami Souissi[2], Jiang-Shiou Hwang[1, 6], Fung Chi Ko[5, 7]*

[1] Institute of Marine Biology, National Taiwan Ocean University, Keelung, Taiwan
[2] University Lille, CNRS, University Littoral Cote d'Opale, UMR 8187, LOG, Laboratoire d'Océanologie et de Géosciences, 62930 Wimereux, France
[3] Argentine Institute of Oceanography, Bahia Blanca, Argentina
[4] National South University, Chemistry Dept., Area III, Bahía Blanca, Argentina
[5] National Museum of Marine Biology and Aquarium, Checheng, Pingtung, Taiwan
[1] Institute of Marine Biology, National Taiwan Ocean University, Keelung, Taiwan
[6] Center of Excellence for the Oceans, National Taiwan Ocean University, Keelung 20224, Taiwan
[7] Institute of Marine Biology, National Dong-Hwa University, Pingtung, Taiwan

Abstract

Sediment profile and mud shrimp (*Austinogebia edulis*) from the coastal wetland of central Taiwan in 2017 and 2018 were analyzed for concentration**,** source, and composition of persistent organic pollutants (POPs) including polycyclic aromatic ~~and~~ hydrocarbon (PAHs), polybrominated diphenyl ethers (PBDEs), organochlorine pesticides (DDT and HCB), and polychlorobiphenyls (PCBs). Results suggest that the determining factor for spatial distribution of POPs might be proximity to contaminant sources near industrial zones. Based on molecular indices, PAHs were substantially of both pyrolytic and petrogenic origins. The main sources for PCBs were Aroclor 1016 and 1260 and ~~the~~ congener BDE 209 was the dominant component among PBDE congeners. The risk assessment on mud shrimp illustrated a borderline threat, with DDT concentrations almost reaching standardized values of Effects Range-Low (ERL). Bioaccumulation factors for DDTs and PCBs (17.33 and 54.59, respectively) were higher than other POPs in this study. Further study is essential to assess and understand the impact of these chemicals on the wetland ecosystem near this heavily industrialized area.

Keywords- POPs; PAHs; PBDEs; PCBs; sediment; mud shrimp

Capsule:
The first in-depth study of POP level and distribution in coastal sediment and benthos at ~~the~~ central Taiwan indicat~~es~~ the potential impact of ~~industrial~~ to the wetland ecosystem.

Correspond~~ed~~ author: ko@gms.ndhu.edu.tw

1. Introduction

Persistent organic pollutants (POPs) are pervasive contaminants found in different domains of the environment (Ramdine et al., 2012; Ahmed et al., 2017; Nascimento et al., 2017; Ramzi et al., 2017), especially the coastal and esturaine sediments due to a rise in industrialization along the coastal regions. Increasing anthropogenic activities, such as discharge of pesticides and fertilizers and incomplete combustion of petrochemicals, have significantly elevated the loading of POPs to the underlying sediment, which has often been considered as the ultimate sink for numerous chemical pollutants (Denis et al., 2012; Bayen, 2012; Cao et al., 2015; Fusi et al., 2016; Tongo et al., 2017; Yuan et al., 2017; Huang et al., 2017). Coastal wetland with intertidal zones are considered to receive the most stress due to the fluctuation of different physical parameters, and are more susceptible to anthropogenic activities (Chapman and Reed, 2006; Wright et al., 2004). Thus, contamination of tidal flats in wetland is a common scenario and measures should be taken to protect the wetland ecosystem.

The vast intertidal flat of coastal zone at Changhua County, Taiwan, represents a complex environmental ecosystem with high biodiversity of crabs, shrimps, fish, and clams. Mud shrimp (*Austinogebia edulis*) is the dominant species in the tidal flat, where they build deep burrows (>1 m) as their habitats. This mud shrimp is prominent in the Changhua County and is considered to be ecologically and commercially essential; hence, it is frequently monitored through different governmental programs. A previous study demonstrated the sensitivity of *A. edulis* to trace metals and alterations to enzymatic activities and physiology caused by higher concentrations of cadmium (Das et al., 2018). During the past decade, the *A. edulis* population has extensively declined for unknown reasons. Since benthic organisms are more likely to get exposed to

contaminants in sediment than in air and water, the sediment is a good index for recording contamination levels. An emerging hypothesis is that *A. edulis* experience higher contaminant stress since their burrows are commonly high in organic matter compared to surrounding sediment and can therefore accumulate more POPs (Das et al., 2017).

Although many POPs such as polychlorinated biphenyls (PCBs), polybrominated diphenyl ethers (PBDEs), and organochlorine pesticides (OCPs) have been prohibited in many countries, they still continue to be reported as toxic chemicals ~~to pose~~ a global threat to the ecosystem. In 1988, after several years of extensive usage and application, the Environmental Protection Administration of Taiwan listed 10 POPs as toxic substances (Liou et al., 2006). Despite this regulation, previous research has demonstrated extensive levels of POPs in Taiwan, including PAHs, PBDEs, and OCPs, mostly in rivers and coastal areas such as Gao-Ping River (Doong et al. 2004, Fang et al., 2008, Jiang et al., 2011), Kenting coral reef (Cheng et al., 2010, 2012), Danshui River and Keelung River (Hung et al., 2006, 2007, 2010, Cheng and Ko, 2018). Given this precedence, there are few studies on POP concentrations in the western coast of central Taiwan, an area heavily impacted by anthropogenic activities (Yeh et al., 2009). In particular, the Changhua coastal industrial park, also known as the Changbin Industrial Park, is a conglomeration of industries, which ~~includes~~ a variety of chemical industries, processors for metals, textile, and food production. Previous research in the area has demonstrated poor air quality with elevated levels of trace metals and PAHs (Chen, et al., 2016; Hsu et al., 2016). However, there is no information on the distribution and effects of POPs in the Changbin Industrial Park.

The aims of this study are to investigate the spatial and temporal distribution of several POPs in sediments and benthic organisms (mud shrimp, *A. edulis*) near Changbin Industrial Park, identify the possible sources of POPs, and assess the potential ecological risks to benthic organisms in this area.

2. Material and methods

2.1 Sample collection

The sediment and biota (mud shrimp) samples from around the industrial area in the western coast of central Taiwan were collected at five stations along the coastline: stations A, B, C, D, and E (Figure 1; Table S1 in supplementary file) from September 2017 to January 2018. The sampling sites experience a wet season (monsoon) from late July until the end of October and a dry season from December until March (winter). Sampling was conducted once in the wet season (September, 2017; temperature of ~28^{0} C) and once in dry season (January, 2018; temperature of ~15^{0} C). The sediment samples were collected by coring with the deepest layer at 50 cm, middle layer at 25 cm, and the surface at 0 cm. Some sediment samples were kept for analysis of total organic carbon (TOC) and grain size. In this study, the mud shrimps *A. edulis* were collected only from station A and E due to their absence from the other stations (B, C, and D) during sampling. All samples were wrapped in aluminum foil, stored in an icebox, and immediately transported to the laboratory. Samples were homogenized on a clean stainless steel tray, transferred to solvent cleaned glass bottles, and stored in -20^{0} C until further analysis.

2.2 Sample Preparation

The preparation and extraction of sediment samples and mud shrimp (*A. edulis*) were

conducted as described by Ko and Baker (1995, 2004). About 5 g of dried sediment and shrimp of each sample were ground finely with anhydrous Na_2SO_4 in a mortar pestle, ~~following~~ by extraction in a Soxhlet apparatus for 24 h with hexane and acetone (1:1; v/v). Every sample was spiked with surrogate standards (*d8*-Napthalene, *d10*-Fluorene, *d10*-Fluoranthene and *d12*-perlene for PAHs and PCB congener14, congener65 and congener166 for PCBs, OCPs, and PBDEs) prior to extraction in order to calculate the percentage of procedural recovery. Activated copper wires or granules were used as necessary to remove elemental sulfur from sediment samples. A rotary vacuum evaporator was used to concentrate the extract. Fractionation and further cleaning of the extract to remove polar interference was performed using 8 g of 6% deactivated alumina packed in a column. Extracts were concentrated using a rotary evaporator followed by a gentle purified nitrogen stream. PAH concentrations were measured by gas chromatography-mass spectrometer (GC–MS) (Varian-320). After PAH analysis, the sample extracts were passed through a glass column packed with 8 g of 2.5% deactivated florisil and covered with about 1 cm Na_2SO_4. The column was conditioned with 35 ml petroleum ether (PE) and dichloromethane (DCM) mixture (1:1) solvent with further addition of 35 ml PE. The PCBs, OCPs, and PBDEs were eluted with 35 ml PE. The eluate was transferred to hexane, concentrated by rotary evaporation, and further dried to less than 0.1 ml under a gentle stream of nitrogen. For quantification, the internal standards (PCB congener30 and congener204 for PCBs and OCPs, and PBDE congener209 for PBDEs) were added to each sample before analyzing the PCB, OCP, and PBDE concentrations with a GC-triple- quadruple mass spectrometry system (TQ-8050, Shimadzu).

2.3 Analytical techniques

The PAHs were determined using a Varian 320 GC-MS, while PCB, DDT, HCB and PBDEs analysis were conducted via GC-MS/MS (TQ-8050, Shimadzu). Separation was performed with a VF-5ms column (30 m, 0.25 mm i.d., 0.25 μm film thicknesses) for PAHs, and OCPs, VF5ht (15 m, 0.25 mm i.d., 0.1 μm film thickness) for all PBDE congeners, and DB-5 (60 m, 0.25 mm i.d., 0.25 μm film thickness) for PCBs. The oven temperature programs for POPs of the instrumental analysis are described in supplementary data. The method detection limits (MDLs), recovery analyzed process, and the quantitative ion and confirm ion in mass spectrometry for each POP are listed in Tables S2, Table S3, and Table S4, respectively.

2.4 Total organic carbon determinations

The total organic carbon (TOC) content in the sediment was determined by using an Elementar Vario EL III (Exeter Analyutical Inc. Germany) after removal of inorganic carbon. (Cheng et al., 2012; Jiang et al., 2011). For each sample, three replicates were used to determine the TOC.

2.5 Quality assurance and quality control

Analysis was conducted according to the standard quality assurance protocols. Glassware and apparatus were washed with detergent and double distilled water, followed by a rinse with acetone and hexane. All analysis included procedural blanks, samples (in duplicate), and spiked samples to assure the quality of the extraction and to detect any transmission of contaminants that might have occurred during analysis. In order to find out the recovery efficiency, four PAH surrogates (*d8*-napthalene, *d10*-fluorene, *d10*-fluoranthene, and *d12*-perylene) and three PCB surrogates (congener14, congener65, and congener166) were added prior to extraction in all the

sample tubes, including the blank. The average recoveries of the standards were 62.8±13.5%, 84.2±10.7%, and 89.9±12.4%, 82.9±18.3% for the PAHs and 73.9±14.5%, 83.3±16.8%, 84.0±17.3% for the PCBs, respectively. The concentrations of POPs were not corrected for surrogate recoveries. The ranges of recovery of spiked individual POPs were 65.3% -100.6% for PAHs, 57.2%-90.6% for PCBs, 64.4%-80.3% for HCB, 64.4%-94.6% for DDTs, and 66.6%-118.8% for PBDEs, respectively. The method detection limits (MDLs) of POPs were defined as the average mass of each compound in the blanks plus three times the standard deviation. The mass of compounds below the MDLs were computed as zero. Quantification of POPs was done by the internal standard method. The relative standard deviation of relative response factor (RRF) was below 10%.

3. Results and Discussion

3.1 Spatial and temporal distribution of POPs

Figure 2a shows the concentration of different POPs in the surface sediment of five sampling stations around Changhua County collected in the wet season (August) and the dry season (January). Among the five POPs series that were analyzed, PAHs, PCBs, DDTs and PBDEs were detected in all the samples except HCBs in station A and station E. Station C had the highest concentration of t-PAHs (332.95 ng/g dw), t-PCBs (4.34 ng/g dw), t-HCBs (0.18 ng/g dw) and t-PBDEs (58.36 ng/g dw) and station D had the highest concentration of t-DDTs (2.63 ng/g dw). Station C and station D had much higher concentrations of all the measured t-POPs, indicating site-specific pollution levels at these stations, probably due to their proximity to Changhua Industrial Park. The POP concentrations in this study did not significantly differ

between seasons. Figure 2b shows the total concentration of all the POPs in the sediment core depths (0 cm, 25 cm, and 50 cm) at the five sampling stations were not significantly different.

3.2 Comparison of POP concentrations with other coastal and estuarine regions in the world

The concentration of PAHs, PCBs, and DDT in the present study was in a range similar to that of Gao-ping estuary in south Taiwan and in Southeast Asian countries, which is substantially lower than in the other coastal areas of the world (Table 1). In this study, the DDT concentrations near the Industrial Park (stations C and D) were significantly higher than in other parts of Changhua County, but much lower when compared to the DDT concentrations found across the globe. In this study we also analyzed the concentration of PBDEs. The t- PBDE concentrations ranged from 0-58.36 ng/g dw. This range of t-PBDEs was similar to the Macao coast in China where the reported range was from 0.6-41.3 ng/g dw (Mai et al. 2005). We found that the discharge of PBDEs in the Industrial Park was also much higher than in other parts of Changhua County, including the southwestern coast (station E) where the t-PBDE concentrations was below the detection limit.

3.3. Compositional profiles

3.3.1. PAHs

PAH isomer ratios have been used to determine PAHs sources and estimate the importance of combustion and petroleum-derived PAHs (Yunker et al., 2002, Budzinsky et al., 1997). The usual index of combustion (anthropogenic) input is an increase in the proportion or molecular mass totals of the less stable or kinetically produced parent PAH isomers relative to

the thermodynamically stable isomers, such as fluoranthene relative to pyrene. Index calculations are traditionally restricted to PAHs within a given molecular mass to minimize factors such as differences in volatility, water/carbon partition coefficients, adsorption, and in most cases closely reflect the source characteristics of PAHs.

PAH ratios considered were primarily proportions of fluoranthene to fluoranthene plus pyrene (Fl/202) and indeno-[*1, 2, 3-cd*]pyrene (IP) to IP plus benzo[ghi]perylene (IP/276) (Figure 3). Fl/202 ratios less than 0.40 indicate petroleum (oil, diesel, and coal) and ratios between 0.40 and 0.50 indicate liquid fossil fuel (vehicle and crude oil) combustion, while ratios over 0.50 indicate grass, wood or coal combustion. Similarly, IP/276 ratios less than 0.20 indicate petroleum, ratios between 0.20 and 0.50 indicate liquid fossil fuel combustion, and ratios over 0.50 indicate grass, wood or coal combustion. Furthermore, these two parent PAH ratios are supplemented by anthracene (An) to An plus phenanthrene (An/178). An/178 ratios less than 0.10 indicate petroleum, while ratios greater than 0.10 indicate combustion.

In this study, sediment samples presented a mean Fl/202 ratio of 0.50 ± 0.03 (n=42) and an IP/276 mean ratio of 0.25 ± 0.12 (n=35) (Figure 3), indicating a pyrogenic source impacting the area. The mean An/178 ratio was 0.07 ± 0.03 (n=42), pointing to petrogenic inputs.

Principal Components Analysis (PCA) was used to extract underlying common factors (principal components, PCs) and analyze relationships among observed variables. As a result of an effective extraction process, PC1 accounted for the major proportion of the total data variance while the second and following PCs progressively explained smaller amounts of data variation. Prior to analysis, values under the method detection limit (MDL) in the data set were replaced with random values under the MDL value.

Concentrations of 40 PAHs as active variables and 42 samples as cases were used. The number of factors extracted from the variables was determined according to Kaiser´s rule, which retains only factors with eigenvalues exceeding 1. As performed in other studies (e.g., Golobocanin et al., 2004), a method of factor rotation to get as many positive loadings as possible to achieve a more meaningful and interpretable solution was preferred (Varimax normalized).

The majority of the variance (88.3%) was explained by three principal components vectors. PC1 accounted for 73.1 % of the total variance, PC2 accounted for 6.2%, while PC3 accounted for 3.9% of the variance. Figure 4a shows the loadings for the individual PAHs at the principal components plot. All the compounds were found to be positively correlated along the PC1 axis; indeed, PC1 had high correlation (r^2=0.7) with alkylated derivatives of PAHs, which are markers of petrogenic origin. This component also included some pyrogenic markers such as fluoranthene, pyrene, benz[*a*]anthracene, chrysene (coal combustion) and even retene, a marker of wood combustion. In addition, PC1 compounds were strongly correlated with the t-PAHs concentration (r^2=0.91), indicating PC1 as mixed origin with over-imposition of petrogenic origin and a quantitative correlation component.

PC2 presented significant positive loadings for two 3-ringed PAHs compounds: fluorene and dibenzothiophene. Fluorene has been reported as a dominant PAH in the coke oven signature (Khalili et al., 1995) while dibenzothiophene (thiophenes in general) is a marker of diesel-powered vehicles (Duval and Friedlander, 1981). Such PAHs are the result of combustion/pyrolitic processes and are absent in crude oil or refined products. Consequently,

PC2 was defined as a pyrogenic component including coke combustion and diesel motors exhaust.

PC3 presented a significant correlation with indeno[*1*, *2*, *3*-*c*,*d*]pyrene, a six fused ring compound, which is a common marker of pyrolysis. The principal components plot (Figure 4a) shows different PAHs clustering. PAHs in the three main clusters may originate from different origin sources. Following PCA, a portion of the total variance of PAH concentrations is explained by source contribution, with petrogenic origin as the prevalent contribution over the sampled area. These results are consistent with our previous findings. Figure 4b shows the 2-D score plot with PC1 and PC2 axes, characterizing the sampling stations according to the first and the second components. In this context, we identified three major groups of samples distributed along the three axes (Figure 4a). PC1-positive coordinates include 19 samples dominated by stations C and D. These samples are located in a petrochemical industrial park wastewater discharge zone, an area defined above as a hotspot because of its total PAHs levels. The remaining samples can be divided into different PC2 and PC3 contributions, with stations B and E mainly correlating with PC2 (coke + diesel combustion) and stations A and B correlating with PC3 (gasoline, coal combustion). In brief, PCA allowed us to assess different PAH sources and classify the sampling sites. These PCA results reflect the conclusions drawn from the ratiometric analysis.

As shown in Figure 4b, stations C and D represented marked petrogenic PAH inputs, concluding that the PAHs sources at the most impacted locations in Changhua County are mainly petrogenic inputs and not combusted oil and petroleum derivatives.

3.3.2. Organochlorines and PCBs

Commercial grade DDT generally contains 90% DDT, 5% DDE, <1% DDD, and <0.5% unidentified compounds (WHO, 1979). DDT-isomers have a long persistence in the environment, gradually degrading to DDE and DDD under both aerobic and anaerobic conditions. In general, the trend of concentrations of DDT and its metabolites present in sediments was DDT>DDD/DDE, indicating quite recent inputs of commercial DDT to the environment. Despite this, different spatial trends were identified: in the north (stations A and B; catchment of city sewage outlet and harbours), there were medium DDT levels (0.17 ng/g dw) and the trend observed for its derivatives was DDT >DDD> DDE. In the south (stations D and E; reserve), the DDT levels were the minimum recorded (0.02-0.06 ng/g d.w), while the industrial stations C and D showed the maximum levels (>0.8 ng/g dw). The dominance of DDTs in stations C and D sediments, as well as the maximum concentration achieved in top layer, indicates slow degradation of DDTs or recent inputs of DDT at these locations (Yuan et al., 2001).

Concentrations of PCBs in worldwide comparison were relatively lower (< 4 ng/g dw). PCB levels were highest in stations C and D, lower in stations A and B, and lowest in station E, reflecting the land use/cover in these areas. The major compounds found in the area were congeners 132,153, 31, 28 and 5+8. Comparing the pattern of percentage of chlorinated compounds with the average known Aroclor mixes (UNEP), we found that the source of PCB contamination in the study area was mixed pattern, involving Aroclor 1016 and Aroclor 1260.

3.3.3. PBDEs

The concentration of total PBDE (Σ10 congeners) ranged from 0-57.6 ng/g dw, with BDE-209 being the most abundant congener in all the stations and in both seasons. The highest concentration of PBDE was found in station C (57.60 ng/g dw) in the deepest layer (50 cm) of sediment in the dry season (January). This result was in accordance with studies in other parts of world which described greater concentrations of POPs in dry seasons than in the wet seasons and higher PBDE concentrations near chemical industries (Doong et al., 2002; Moon et al., 2007; Cheng and Ko, 2018). BDE-209, the most abundant congener, composed almost 85-90% of the total PBDE concentration in all the stations in both seasons. The highest BDE-209 concentrations were recorded in station C (57.60 ng/g dw at 50cm in dry season and 47.89 ng/g dw at 25 cm in wet season). Similar results have been published by previous studies, where BDE 209 was found to be the most abundant congener, accounting for almost 90-100% of the composition of t-PBDE and showing highly variable concentrations throughout the world (Voorspoels et al., 2004; Moon et al., 2007; Cheng and Ko, 2018). The observation is corroborated by recent studies demonstrating the high commercial usage of Br-10 mixtures in Taiwan. The low brominated BDEs found in the top layer of each station might be the product of debromination of higher brominated congeners of PBDE.

3.4. Concentrations of POPs related with sediment characteristics

The total organic carbon (TOCs) of sediment is often considered as a prime factor for the distribution of POPs in a particular area and are widely compared in studies related to organic contaminants (Hung et al., 2006; 2007; 2010; Yang et al., 2011; Gao et al., 2015; Cheng and Ko, 2018). Persistence of POPs in aquatic sediments is due to their low rate of degradation and vaporization, low water solubility, and high partitioning to particles and organic carbon. To test

this in the present study, a correlation between each POP level and % TOC was assessed in this study (Figure 5).

The TOCs of the sediments ranged from 0.16%- 0.93%. The lowest TOC content was recorded in station A and the highest in station C, both in the middle layer (25cm) of the sediment column. There were strong and significant correlations between each POP (t-PAH, t-PCB, t-DDT and t-PBDE) with TOC (Figure 5). Previous studies have reported similar findings when comparing TOC and POP concentrations (Doong et al., 2008; Lee et al., 2014; Wang et al., 2015; Zhao et al., 2012, Cheng and Ko, 2018). Hence, TOC content can be considered as a useful tool to assess or surveil the concentrations of diverse organic contaminants in sediments (Doong et al., 2008; Lee et al., 2014; Cheng and Ko, 2018).

3.5. Ecological risk assessments of POPs in sediments

Taiwan still lacks a standard for acceptable concentrations of individual POPs in seafood and sediment. However, previous studies have used the general rule presented by Long et al., 1995 from the Canadian councils of Ministers of the Environment (CCME, 2002), which uses the Threshold Effect Level (TEL) and the Probable Effect Level (PEL) to estimate the risk of POPs in sediments and benthic organisms. This standard criterion represents the limit above which (50% frequency, “Effects Range-Median”) contaminant concentration will be considered toxic and below which (10% frequency, “Effects Range-Low”), contaminant concentrations will rarely cause detrimental effects. Using these sediment quality guidelines (SQG) set by Long et al., 1995, none of the POP concentrations at the stations exceed the ERL values (Table. 2). However the t-DDT concentrations in stations C and D are closer to the standardized ERL values.

This can be explained by the use of pesticides for nearby agriculture and the proximity of these two stations to the Industrial Park. Also, the concentration of t-PAHs and t-PCBs are higher in stations C and D than in the other stations, which may have caused the absence of mud shrimps in these sites. Therefore the concentrations of POPs in the wetland sediment of this study, including the Changhua Industrial Park, likely have rudimentary or marginal effects on benthic organisms.

3.6 Bioaccumulation of POPs in the mud shrimp

Austinogebia edulis is a common seafood consumed by locals of the western coast of Taiwan and it has high economic importance. The concentration of each measured POP in *A. edulis* has been listed in Table 3. Out of the five stations (A, B, C, D, and E) only two stations (A and E) showed the presence of mud shrimps. The concentration of t-PAH was higher when compared to the other POP concentrations. HCB was not detected in any of the samples that were tested. The concentration of all the POPs tested were found to be much higher in station E than station A. Since the Taiwanese government has declared station A a restricted mud shrimp conservation area, the level of contamination by organic pollutants are comparatively lower than ~~in~~ surrounding unrestricted and open areas.

In order to implement a tool for the regulation of contaminated habitats, and for the assessment of potential risk to benthic animals, calculation of bioaccumulation factors (BAFs) have been proved to be a useful approach (Parkerton, et al., 1993; Nakata et al., 2003). The BAFs for each pollutant in stations A and E was calculated by using the equation (1) and listed in Table 3.

$$\text{BAFs} = \frac{\text{Concentration of POPs in mud shrimp}}{\text{Concentration of POPs in sediment}} \quad \ldots\ldots\ldots\ldots (1)$$

The BAFs in the mud shrimp *A. edulis* were much higher in station E than in station A, especially for t-PCB and t-DDT (Table 3). *Austinogebia edulis* are burrowers in the muddy substratum and are get exposed to the sediment column throughout their entire lifespan. Previous literatures have documented that BAFs values are highly variable and dependent on various parameters, like direct contact or exposure of the organisms with the sediment column, amount of the residue present in the sediment etc. (Hawker, et al., 1985; Froese et al., 1998; Nakata et al., 2003; Iannuzzi et al., 2011; Chang, 2017). Thus, even a hydrophobic compound like PCB may have a higher BAF value in the body of a burrowing mud shrimp. Previous literature noted ranges of BAFs for PAHs to be higher in certain crabs and lugworms than in oysters and clams (Nakata et al., 2003). Several studies have accounted for varying BAFs for PCB and PBDE in fishes, crabs, mussels, shrimps, etc. For instance, the Chinese Mitten Crab exhibited the highest value of Biota-Sediment Accumulation Factor (BSAF) for PCBs (2900), followed by the shore crab in the Scheldt estuary of Netherlands–Belgium (2330; Van Ael et al., 2012). In the same site the value of PCB BSAF in the brown shrimp (606), blue mussels (1090), and worms (1180) were found to be comparatively lower (Van Ael et al., 2012). However, the PCB BSAF for the Blue crab (0.22-1.7) and the white perch (0.34-1.5) in the Passaic River, USA accounted for minute values of bioaccumulation factor (Iannuzzi et al., 2011). The BSAF for PBDEs in the Scheldt estuary was also higher in Chinese mitten crab (2520) and shore crab (598), but in comparison was much lower in brown shrimp (162) and blue mussels (304). The PBDE BSAF found in the northern horse mussel of the Vancouver Island, Canada varied from 0.95- 527 (deBruyn et al.,

2009). The present study found lower BAF values for each measured POPs in mud shrimps when compared to other global studies. The variations in the results can be accounted for by different trophic levels or the modes of ingestion amongst the benthic biota. For instance, lower molecular weight PAHs such as phenanthrene and anthracene have been reported to absorb compounds from the interstitial water directly, whereas higher molecular weight compounds adsorb on particulate matter (Baumard et al., 1998; Nakata et al., 2003). Thus, the bioaccumulation pathways for different aquatic and intertidal organisms differ greatly depending on the molecular weight of the compounds, modes of ingestion, absorption through skin, and the ability of the organisms to metabolize the compounds.

4. Conclusion

This study presents the first comprehensive survey of PAHs, PBDEs, organochlorine pesticides, and PCBs in sediments from Changhua County, Taiwan providing useful information on concentrations, composition and sources. The spatial distribution of POPs showed that proximity to sources was the most important determining factor for the distribution of these contaminants. In general, POP concentrations were greater in samples collected near the industrial area (C and D) than those from the non-industrial locations (A, B and E). Molecular indices such as Fl/202, IP/276, and An/178 revealed the existence of both pyrolitic and petrogenic inputs at the area. Further, the use of PCA enabled the classification of sampling sites in accordance to their main source of PAHs. Considering PCBs, Aroclor 1016 and 1260 were assessed as the main technical sources for the area. Although levels were below scientific sediment guidelines, the study identified recent DDT inputs to the area. Beyond the

anthropogenic impact on the sediment, POPs appear to pose a potential rudimentary or marginal risk in regards to their effect on benthic organisms.

Acknowledgement

We acknowledge a grant of the Ministry of Science and Technology (MOST) of Taiwan for financial support through grants: MOST 104-2611-M-019- 004, MOST 105-2621-M-019 001, MOST 105-2918-I-019-001, MOST 106-2621-M-019- 001, and MOST 107-2621-M-019 001. We specially thank Mr. Chih-Ming Lin and Dr. Shao Hung Peng for their assistance during the field sampling. This paper is a contribution to the International Joint Laboratory between Lille University, France and National Taiwan Ocean University.

Table 1. . Comparison of the POP concentrations (ng/g dw) in the sediments from the other coastal areas with the present study.

Locations	t-PAH (ng/g dw)	t-PCB (ng/g dw)	t-DDT (ng/g dw)	Reference
**Stations A, B, and E**	**19.96-129.66**	**0.04-1.11**	**0.03-0.21**	**This study**
**Stations C and D** (Changbin Industrial Park)	**135.15-332.95**	**0.24-4.34**	**0.106- 2.63**	**This study**
Pearl River Estuary, China	138–1100	-	1.38–25.4	Chen et al., 2006
Yangtze Estuarine, China	32.10–71.10	ND–63	ND–5.10	Adeleye et al., 2016
Victoria Harbor, Hong Kong		3.2–27	1.4–30	Connell et al., 1998
Coastal region, Singapore	12.65-93.85	1.40–330	3.40–46.10	Wurl and Obbard, 2005
Coast of Korea	9.1-1400	0.17–371	0.01–135	Hong et al., 2006
Gao-ping Estuaries, Taiwan	1.43–356	0.38–5.90	0.44–1.88	Doong et al., 2008
Northeastern coast of India	**-**	0.18–2.33	0.18–1.93	L. Guzzella et al., 2005
Bay of Bengal, India	20.35-2615.38	0.02–6.57	0.04–4.79	Binelli et al., 2008
West coast of India	**-**	**-**	1.47–25.17	Sarkar et al., 1997
Bay of Biscay, France	0.7–300	ND–375	**-**	Bartolomé et al., 2006
The Baltic Sea	9.5-1900	0.01–6.20	0.13–0.50	Pikkarainen, 2007
Cantabrian Sea, Spain	19–2123	ND–160	4.2–25	Sánchez-Avila et al., 2013
San Francisco Bay, CA	2944–29590	-	11-23330	Pereira et al., 1996
Guánica Bay, Puerto Rico, USA	0.64–4663	14 0.11–3059.90	0.00–69.25	Whitall et al., 2014
Bahia Blanca Estuary, Argentina	15-10260	0.61-17.6	ND-2.3	Tombesi et al, 2016

Table. 2 Comparison of standard values set for toxicity (SQG and CCME values ng/g dw) with the POP concentrations in the sediments from this study.

	SQG (Long et al., 1995) and CCME (2002)				This study				
					Stations				
	**ERM**	**ERL**	**PEL**	**TEL**	**A**	**B**	**C**	**D**	**E**
**t-PAHs**	44792	4022	6676	655	58.42	99.20	238.93	254.38	41.80
**t-PCBs**	180	22.7	189	22	0.25	0.73	2.07	1.75	0.07
**t-DDTs**	46.1	1.58	4.77	3.89	0.06	0.16	0.67	0.79	0.06

Table 3. The total concentrations and bioaccumulation factors (BAFs) of POPs in mud shrimps.

**Station**	**t-PAHs**	**t-PCBs**	**t-PBDEs**	**t-DDTs**	**HCB**
**Concentrations (ng/g)**					
**Station A**	61.9	0.5	1.2	0.4	nd
**Station E**	94.1	3.9	2.2	1.1	nd
**Bioaccumulation factor**					
**Station A**	1.1	1.9	0.5	6.2	
**Station E**	2.3	54.6	0.9	17.3	

nd: lower than MDLs

Figure Caption

Figure 1. Map showing the sampling stations (A, B, C, D and E) at the western coast of Taiwan.

Figure 2. Total concentrations of POPs in different stations (A, B, C, D, E); a. Seasonal variation. b. distribution at different depths (0 cm, 25 cm, and 50 cm).

Figure 3. Comparison of selected PAHs ratios for 42 sediment samples along the area of study. Abbreviations refer to the ratios of fluoranthene plus pyrene (Fl/202), indeno-[*1, 2, 3-cd*]pyrene (IP) to IP plus benzo[*ghi*]perylene (IP/276) and anthracene (An) to An plus phenanthrene (An/178).

Figure 4a. The PCA loading plot of sedimentary PAHs; b. Score plot illustrating the distribution of PAHs compounds in the sampled areas along PC1 and PC2 axis

Figure 5. The relationship between TOC (%) and t-PAH, t-PCB, t-DDT, and t-PBDE in ng/g dw.

References

Adeleye A.O., Jin, H., Di, Y., Li, D., Chen, J., Ye, Y., 2016. Distribution and ecological risk of organic pollutants in the sediments and seafood of Yangtze Estuary and Hangzhou Bay, East China Sea. Sci. Total Environ. 541, 1540–1548. https://doi.org/10.1016/j.scitotenv.2015.09.124

Adeogun, A.O., Onibonoje, K., Ibor, O.R., Omiwole, R.A., Chukwuka, A.V., Ugwumba, A.O., Ugwumba, A.A.A., Arukwe, A., 2016. Endocrine-disruptor molecular responses, occurrence of intersex and gonado-histopathological changes in *Tilapia* species from a tropical freshwater Dam (Awba Dam) in Ibadan, Nigeria. Aquat. Toxicol. 174, 10–21. https://doi.org/10.1016/j.aquatox.2016.02.002

Ahmed, M.M., Doumenq, P., Awaleh, M.O., Syaktib, A.D., Asia, L., Chiron, S., 2017. Levels and sources of heavy metals and PAHs in sediment of Djibouti-city (Republic of Djibouti). Mar. Pollut. Bull. 120, 340–346. https://doi.org/10.1016/j.marpolbul.2017.05.055

Allchin, C.R., Law, R.J., Morris, S., 1999. Polybrominated diphenylethers in sediments and biota downstream of potential sources in the UK. Environ. Pollut. 105, 197– 207. https://doi.org/10.1016/S0269-7491(98)00219-X

Arias, A. H., Spetter, C. V., Freije, R. H., Marcovecchio, J. E., 2009. Polycyclic aromatic hydrocarbons in water, mussels (*Brachidontes* sp., *Tagelus* sp.) and fish (*Odontesthes* sp.) from Bahía Blanca Estuary, Argentina. Estuar Coast Shelf Sci. 85(1), 67–81. 10.1016/j.ecss.2009.06.008

Arias, A. H., Vazquez-Botello, A., Tombesi, N., Ponce-Vélez, G., Freije, H., Marcovecchio, J., 2010. Presence, distribution, and origins of polycyclic aromatic hydrocarbons (PAHs) in sediments from Bahía Blanca estuary, Argentina. Environ Monit Assess. 160(1-4), 301. https://doi.org/10.1007/s10661-008-0696-5

Aris, A.Z., Shamsuddin, A.S., Praveena, S.M., 2014. Occurrence of 17α-ethynylestradiol (EE2) in the environment and effect on exposed biota: a review. Environ. Int. 69, 104–119. https://doi.org/10.1016/j.envint.2014.04.011

Bartolomé, L., Tueros, I., Cortazar, E., Raposo, J.C., Sanz, J., Zuloaga, O., de Diego, A., Etxebarria, N., Fernández, L.A., Madariaga, J.M., 2006. Distribution of trace organic contaminants and total mercury in sediments from the Bilbao and Urdaibai Estuaries (Bay of Biscay). Mar. Pollut. Bull. 52, 1111–111. 10.1016/j.marpolbul.2006.05.024

Baumard, P., Budzinski, H., Garrigues, P., 1998. Polycyclic aromatic hydrocarbons in sediments and mussels of the western Mediterranean sea. Environ. Toxicol. Chem. 17(5), 765–776.10.1002/etc.5620170501

Bayen, S., 2012. Occurrence, bioavailability and toxic effects of trace metals and organic contaminants in mangrove ecosystems: a review. Environ. Int. 48, 84–101. https://doi.org/10.1016/j.envint.2012.07.008

Bergman, A., Heindel, J.J., Kasten, T., Kidd, K.A., Jobling, S., Neira, M., Zoeller, R.T., Becher, G., Bjerregaard, P., Bornman, R., Brandt, I., Kortenkamp, A., Muir, D., Drisse, M.N.B., Ochieng, R., Skakkebaek, N.E., Byléhn, A.S., Iguchi, T., Toppari, J., Woodruf, T.J., 2013. The Impact of Endocrine Disruption: A Consensus Statement on the State of the Science. Environ. Health Perspect. 121, 104–106. https://doi.org/10.1289/ehp.1205448

Bin, J., Hai-long, Z., Guo-qiang, H., Hui, D., Xin-Gang, L., Hong-Tu, S., Rui, L., 2007. Characterization and distribution of polycyclic aromatic hydrocarbon in sediments of Haihe River, Tianjin, China. J Environ Sci. 19, 306–311. https://doi.org/10.1016/S1001-0742(07)60050-3

Binelli, A., Sarkar, S.K., Chatterjee, M., Riva, C., Parolini, M., Bhattacharya, B., Bhattacharya, A.K., Satpathy K.K., 2008. A comparison of sediment quality guidelines for toxicity assessment in the Sunderban wetlands (Bay of Bengal, India). Chemosphere 73, 1129–1137. https://doi.org/10.1016/j.chemosphere.2008.07.019

Budzinski, H., Jones, I., Bellocq, J., Piérard, C., Garrigues, P., 1997. Evaluation of sediment contamination by polycyclic aromatic hydrocarbons in the Gironde estuary. Mar Chem. 58(1-2), 85–97. 10.1016/s0304-4203(97)00028-5

Cao, Q.M., Wang, H., Qin, J.Q, Chen, G.Z., Zhang, Y.B., 2015. Partitioning of PAHs in pore water from mangrove wetlands in Shantou, China. Ecotoxicol. Environ. Saf. 111, 42–47. https://doi.org/10.1016/j.ecoenv.2014.09.023

Carvalho, P. N., Rodrigues, P. N. R., Basto, M. C. P., Vasconcelos, M. T. S. D., 2009. Organochlorine pesticides levels in Portuguese coastal areas. Chemosphere. 75(5), 595–600.10.1016/j.chemosphere.2009.01.060

Casatta, N., Mascolo, G., Roscioli, C., Viganò, L., 2016. Tracing endocrine disrupting chemicals in a coastal lagoon (Sacca di Goro, Italy): Sediment contamination and bioaccumulation in Manila clams. Sci. Total Environ. 514, 214–222. https://doi.org/10.1016/j.scitotenv.2014.12.051

CCME., 2002. Guidance on the site-specific application of water quality guidelines in Canada: procedures for deriving numerical water quality objectives. Winnipeg, Manitoba.

Chang, G.R., 2017. Persistent organochlorine pesticides in aquatic environments and fishes in Taiwan and their risk assessment. Environ Sci Pollut Res Int. 25(8), 7699–7708. 10.1007/s11356-017-1110-z

Chau, K.W., 2006. Persistent organic pollution characterization of sediments in Pearl River estuary. Chemosphere 64, 1545–1549. https://doi.org/10.1016/j.chemosphere.2005.11.060

Chen, C.F., Chen, C.W., Ju, Y.R., & Dong, C.D., 2016. Vertical profile, source apportionment, and toxicity of PAHs in sediment cores of a wharf near the coal-based steel refining industrial zone in Kaohsiung, Taiwan. Environ Sci Pollut Res Int. 23, 4786–4796. https://doi.org/10.1007/s11356-015-5716-8

Chen, Y.C., Chiang, H.C., Hsu, C.Y., Yang, T.T., Lin, T.Y., Chen, M.J., Chen, N.T., Wu, Y.S., 2016. Ambient $PM_{2.5}$-bound polycyclic aromatic hydrocarbons (PAHs) in Changhua County, central Taiwan: Seasonal variation, source apportionment and cancer risk assessment. Environ Pollut. 218:372-382. 10.1016/j.envpol.2016.07.016.

Cheng, J.O., Cheng, Y.M., Chen, T.H., Hsieh, P.C., Fang, M.D., Lee, C.L., Ko, F.C., 2010. A preliminary assessment of polycyclic aromatic hydrocarbon distribution in the Kenting Coral Reef waters of Southern Taiwan. Arch. Environ. Contam. Toxicol. 58, 489-98. https://doi.org/10.1007/s00244-009-9411-y

Cheng, J.O., Ko, F.C., Li, J.J., Chen, T.H., Cheng, Y.M., Lee, C.L., 2012. Concentrations of polycyclic aromatic hydrocarbon in the surface sediments from inter tidal areas of Kenting coast, Taiwan. Environ. Monit. Assess. 184, 3481–3490. https://doi.org/10.1007/s10661-011-2202-8

Cheng, J.O., Ko, F.C., 2018. Occurrence of PBDEs in surface sediments of metropolitan rivers: Sources, distribution pattern, and risk assessment. Sci. Total Environ. 637–638, 1578–1585. https://doi.org/10.1016/j.scitotenv.2018.05.075

Chapman, P., Reed, D., 2006. Advances in coastal habitat restoration in the northern Gulf of Mexico. Ecol Eng. 26, 1–5. 10.1016/j.ecoleng.2005.09.003

Cleemann, M., Rigetm, F., Paulsen, G.B., Klungsøyr, J., Dietz, R., 2000. Organochlorines in Greenland marine fish, mussels, and sediments. Sci. Total Environ. 245, 87–102. https://doi.org/10.1016/S0048-9697(99)00435-0

Connell, D.W., Wu, R.S.S., Richardson, B.J., Leung, K., Lam, P.S.K., Connell, P.A., 1998. Fate and risk evaluation of persistent organic contaminants and related compounds in Victoria Harbour, Hong Kong. Chemosphere. 36, 2019-2030. https://doi.org/10.1016/S0045-6535(97)10087-X

Das, S., Tseng, L.C., Wang, L., Hwang, J.S., 2017. Burrow characteristics of the mud shrimp *Austinogebia edulis*, an ecological engineer causing sediment modification of a tidal flat. PLoS ONE. 12(12): e0187647. https://doi.org/10.1371/journal.pone.0187647

Das, S., Tseng, L.C., Chou, C., Wang, L., Souissi, S., Hwang J.S., 2018. Effects of cadmium exposure on antioxidant enzymes and histological changes in the mud shrimp *Austinogebia edulis* (Crustacea: Decapoda). Environ Sci Pollut Res Int. https://doi.org/10.1007/s11356-018-04113-x

De Bruyn, A.M.H., Meloche, L.M., Lowe, C.J., 2009. Patterns of bioaccumulation of polybrominated diphenyl ether and polychlorinated biphenyl congeners in marine mussels. Environ Sci Technol. 43, 3700–4. 449. doi:10.1016/j.ibiod.2008.12.005

De Castro-Català, N., Kuzmanovic, M., Roig, N., Sierra, J., Ginebreda, A., Barceló, D., Pérez, S., Petrovic, M., Picó, Y., Schuhmacher, M., Muñoz, I., 2016. Ecotoxicity of sediments in rivers: Invertebrate community, toxicity bioassays and the toxic unit approach as complementary assessment tools. Sci. Total Environ. 540, 297–306.

https://doi.org/10.1016/j.scitotenv.2015.06.071

Denis, E.H., Toney, J.L., Tarozo, R., Anderson, R.S., Roach, L.D., Huang, Y., 2012. Polycyclic aromatic hydrocarbons (PAHs) in lake sediments record historic fire events: validation using HPLC-fluorescence detection. Org. Geochem. 45, 7–17. https://doi.org/10.1016/j.orggeochem.2012.01.005

Dercová, K., Šeligová, J., Dudášová, H., Mikulášová, M., Šilhárová, K., Tóthová, L., Hucko, P., 2009. Characterization of the bottom sediments contaminated with polychlorinated biphenyls: Evaluation of ecotoxicity and biodegradability. Int. Biodeterior. Biodegradation. 63(4), 440-449.10.1016/j.ibiod.2008.12.005

Doong, R.A., Peng, C.K., Sun, Y.C., Liao, P.L., 2002. Composition and distribution of organochlorine pesticide residues in surface sediments from Wu-shi River estuary, Taiwan. Mar Pollut Bull. 45, 246–53. https://doi.org/10.1016/S0025-326X(02)00102-9

Doong, R.A., Lin, Y.L., 2004. Characterization and distribution of polycyclic aromatic hydrocarbon contaminations in surface sediment and water from Gao-ping River, Taiwan. Water Res. 38, 1733–1744. https://doi.org/10.1016/j.watres.2003.12.042

Doong, R.A., Lee, S.H., Lee, C.C., Sun, Y.C.,Wu, S.C., 2008. Characterization and composition of heavy metals and persistent organic pollutants in water and estuarine sediments from Gao-ping River, Taiwan. Mar. Pollut. Bull. 57, 846–857. https://doi.org/10.1016/j.marpolbul.2007.12.015

Duval, M.M., Friedlander, S.K., 1981. Source resolution of polycyclic aromatic hydrocarbons in the Los Angeles atmosphere: application of a chemical species balance method with first order chemical decay. Final report Jan-Dec 80. United States.

Eljarrat, E., de la Cal, A., Raldua, D., Duran, C., Barcelo, D., 2004. Occurrence and bioavailability of Polybrominated Diphenyl ethers and Hexabromocyclododecane in sediment and fish from the Cinca River, a tributary of the Ebro River (Spain). Environ. Sci. Technol. 38, 2603–2608. 10.1021/es0301424

Fang, M.D., Ko, F.C., Baker, J.E., Lee, C.L., 2008. Seasonality of diffusive exchange of polychlorinated biphenyls and hexachlorobenzene across the air–sea interface of Kaohsiung Harbor, Taiwan. Sci. Total Environ. 407, 548–565. https://doi.org/10.1016/j.scitotenv.2008.09.021

Feng, K., Yu, B.Y., Ge, D.M., Wong, M.H., Wang, X.C., Cao, Z.H., 2003. Organo-chlorine pesticide (DDT and HCH) residues in the Taihu Lake Region and its movement in soil–water system. I. Field survey of DDT and HCH residues in ecosystem of the region. Chemosphere. 50, 683–687. https://doi.org/10.1016/S0045-6535(02)00204-7

Frignani, M., Bellucci, L. G., Favotto, M., Albertazzi, S., 2005. Pollution historical trends as recorded by sediments at selected sites of the Venice Lagoon. Environ Int. 31(7), 1011–1022.10.1016/j.envint.2005.05.011

Froese, K. L., Verbrugge, D. A., Ankley, G. T., Niemi, G. J., Larsen, C. P., Giesy, J. P., 1998. Bioaccumulation of polychlorinated biphenyls from sediments to aquatic insects and tree swallow eggs and nestlings in Saginaw Bay, Michigan, USA. Environ Toxicol Chem. 17(3), 484–492. 10.1002/etc.5620170320

Fowler, P.A., Bellingham, M., Sinclair, K.D., Evans, N.P., Pocar, P., Fischer, B., Schaedlich, K., Schmidt, J.S., Amezaga, M.R., Bhattacharya, S., Rhind, S.M., O'Shaughnessy, P.J., 2012. Impact of endocrine-disrupting compounds (EDCs) on female reproductive health. Mol. Cell. Endocrinol. 355, 231–239. https://doi.org/10.1016/j.mce.2011.10.021

Fusi, M., Beone, G.M., Suciu, N.A., Sacchi, A., Trevisan, M., Capri, E., Daffonchio, D., Din, N., Dahdouh-Guebas, F., Cannicci, S., 2016. Ecological status and sources of anthropogenic contaminants in mangroves of the Wouri River estuary (Cameroon). Mar. Pollut. Bull. 109, 723–733. https://doi.org/10.1016/j.marpolbul.2016.06.104

Galassi, S., Guzzella, L., Battegazzore, M., Carrieri, A., 1994., Biomagnification of PCBs, p,p′-DDE, and HCB in the River Po ecosystem (Northern Italy). Ecotoxicol. Environ. Saf. 29(2), 174–186.10.1016/0147-6513(94)90018-3

Gao, S., Chen, J., Shen, Z., Liu, H., Chen, Y., 2013. Seasonal and spatial distributions and possible sources of polychlorinated biphenyls in surface sediments of Yangtze Estuary, China. Chemosphere. 91, 809–816. https://doi.org/10.1016/j.chemosphere.2013.01.085

Golobočanin, D. D., Škrbić, B. D., Miljević, N. R., 2004. Principal component analysis for soil contamination with PAHs. Chemometr Intell Lab Syst. 72(2), 219–223. 10.1016/j.chemolab.2004.01.017

Guzzella, L., Roscioli, C., Vigano,` L., Saha, M., Sarkar, S.K., Bhattacharya, A., 2005. Evaluation of the concentration of HCH, DDT, HCB, PCB and PAH in the sediments along the lower stretch of Hugli estuary, West Bengal, northeast India. Environ Int. 31, 523–534. https://doi.org/10.1016/j.envint.2004.10.014

Hawker, D. W., Connell, D. W., 1985. Relationships between partition coefficient, uptake rate constant, clearance rate constant and time to equilibrium for bioaccumulation. Chemosphere. 14(9), 1205–1219. 10.1016/0045-6535(85)90142-0

Hong, H., Xu, L., Zhang, L., Chen, J.C., Wong, Y.S., Wan, T.S.M., 1995. Special guest paper: environmental fate and chemistry of organic pollutants in the sediment of Xiamen and Victoria Harbours. Mar. Pollut. Bull. 31, 229–236. https://doi.org/10.1016/0025-326X(95)00115-4

Hong, S.H., Yim, U.H., Shim, W.J., Li, D.H., Oh, J.R., 2006. Nationwide monitoring of polychlorinated biphenyls and organochlorine pesticides in sediments from coastal environment of Korea. Chemosphere 64, 1479–1488. https://doi.org/10.1016/j.chemosphere.2005.12.056

Hong, W.-J., Jia, H., Li, Y.-F., Sun, Y., Liu, X., Wang, L., 2016. Polycyclic aromatic hydrocarbons (PAHs) and alkylated PAHs in the coastal seawater, surface sediment and oyster from Dalian, Northeast China. Ecotoxicol. Environ. Saf. 128, 11–20. https://doi.org/10.1016/j.ecoenv.2016.02.003

Hsu, C.Y., Chiang, H.C., Lin, S.L., Chen, M.J., Lin, T.Y., Chen, Y.-C., 2016. Elemental characterization and source apportionment of PM10 and PM2.5 in the western coastal area of central Taiwan. Sci. Total Environ. 541, 1139-1150. https://doi.org/10.1016/j.scitotenv.2015.09.122

Huang, Y., Liu, M., Wang, R., Khan, S.K., Gao, D., Zhang, Y., 2017. Characterization and source apportionment of PAHs from a highly urbanized river sediments based on land use analysis. Chemosphere 184, 1334–1345. https://doi.org/10.1016/j.chemosphere.2017.06.117

Hung, C.C., Gong, G.C., Jiann, K.T., Yeager, K.M., Santschi, P.H., Wade, T.L., Sericano, J.L., Hsieh, H.L., 2006. Relationship between carbonaceous materials and polychlorinated biphenyls (PCBs) in the sediments of the Danshui River and adjacent coastal areas, Taiwan. Chemosphere. 65, 1452–146. https://doi.org/10.1016/j.chemosphere.2006.04.037

Hung, C.-C., Gong, G.-C., Chen, H.-Y., Hsieh, H.-L., Santschi, P.H., Wade, T.L., Sericano, J.L., 2007. Relationships between pesticides and organic carbon fractions in sediments of the Danshui River estuary and adjacent coastal areas of Taiwan. Environ. Pollut. 148, 546–554. https://doi.org/10.1016/j.envpol.2006.11.036

Hung, C.-C., Gong, G.-C., Ko, F.-C., Chen, H.-Y., Hsu, M.-L.,Wu, J.-M., Peng, S.-C., Nan, F.-H., Yeager, K.M., Santschi, P.H., 2010. Relationships between persistent organic pollutants and carbonaceous materials in aquatic sediments of Taiwan. Mar. Pollut. Bull. 60, 1010–1017. https://doi.org/10.1016/j.marpolbul.2010.01.026

Hussain, K., Hoque, R.R., 2015. Seasonal attributes of urban soil PAHs of the Brahmaputra Valley. Chemosphere. 119, 794–802. 10.1016/j.chemosphere.2014.08.02

Iannuzzi, J., Butcher, M., Iannuzzi, T., 2011. Evaluation of potential relationships between chemical contaminants in sediments and aquatic organisms from the lower Passaic River, New Jersey, USA. Environ Toxicol Chem. 30: 1721–8.

Jergentz, S., Mugni, H., Bonetto, C., Schulz, R., 2004. Runoff-Related Endosulfan Contamination and Aquatic Macroinvertebrate Response in rural Basins near Buenos Aires, Argentina. Arch. Environ. Contam. Toxicol. 46(3). 10.1007/s00244-003-2169-8

Jiang, J.-J., Lee, C.-L., Fang, M.-D., Ko, F.-C., Baker, J.E., 2011. Polybrominated diphenyl ethers and polychlorinated biphenyls in sediments of Southwest Taiwan: regional characteristics and potential sources. Mar. Pollut. Bull. 62, 815–823. https://doi.org/10.1016/j.marpolbul.2010.12.019

Katsoyiannis, A., 2006. Occurrence of polychlorinated biphenyls (PCBs) in the Soulou stream in the power generation area of Eordea, northwestern Greece. Chemosphere 65, 1551–1561. https://doi.org/10.1016/j.chemosphere.2006.04.004

Keith, L., Telliard W., 1979. ES&T Special Report: Priority pollutants: I-a perspective view. Environ. Sci. Technol.13 (4), 416–423. 10.1021/es60152a601.

Kennish, M. J., 1992. Ecology and Estuaries: Anthropogenic Effects. CRC Press, Boca Raton, Florida.

Keshavarzifard, M., Zakari, M. P., Reza, S., 2017a. Ecotoxicological and health risk assessment of polycyclic aromatic hydrocarbons (PAHs) from consumption of shortneck clam (*Paphia undulata*), and contaminated sediment exposure, Malacca Strait, Malaysia. Arch. Environ. Contam. Toxicol. 10.1007/ s00244-017-0410-0.

Keshavarzifard, M., Zakaria, M. P., Hwai, T. S., 2017b. Bioavailability of polycyclic aromatic hydrocarbons (PAHs) to short-neck clam (*Paphia undulata*) from sediment matrices in mudflat areas of West coast of Peninsular Malaysia. Environ Geochem Health.10.1007/s10653-016-9835-z.

Khalili, N. R., Scheff, P. A., Holsen, T. M., 1995. PAH source fingerprints for coke ovens, diesel and, gasoline engines, highway tunnels, and wood combustion emissions. Atmos. Environ. 29(4), 533–542. 10.1016/1352-2310(94)00275-p

Kim, G.B., Maruya, K.A., Lee, R.F., Lee, J.H., Koh, C.H., Tanabe, S., 1999. Distribution and sources of polycyclic aromatic hydrocarbons in sediments from Kyeonggi Bay, Korea. Mar Pollut Bull. 38:7–15. https://doi.org/10.1016/S0025-326X(99)80006-X

Ko, F.C., Baker, J.E., 1995. Partitioning of hydrophobic organic contaminants to resuspended sediments and plankton in the mesohaline Chesapeake Bay. Mar Chem. 49, 171–188. https://doi.org/10.1016/0304-4203(94)00064-K

Ko, F.C., Baker, J.E., 2004. Seasonal and annual loads of hydrophobic organic contaminants from the Susquehanna River Basin to the Chesapeake Bay. Mar Pollut Bull. 48, 840–851. https://doi.org/10.1016/j.marpolbul.2003.10.014

Konat, J., Kowalewska, G., 2001. Polychlorinated biphenyls (PCBs) in sediments of the southern Baltic Sea — trends and fate. Sci Total Environ. 280(1-3), 1–15. 10.1016/s0048-9697(01)00785-9

Kucklick, J. R., Sivertsen, S. K., Sanders, M., Scott, G. I., 1997. Factors influencing polycyclic aromatic hydrocarbon distributions in South Carolina estuarine sediments. J. Exp. Mar. Biol. Ecol. 213(1), 13–29. 10.1016/s0022-0981(97)00007-5

Lacorte, S., Guillamón, M., Martínez, E., Viana, P., Barceló, D., 2003. Occurrence and specific

congener profile of 40 Polybrominated Diphenyl ethers in river and coastal sediments from Portugal. Environ. Sci. Technol. 37, 892–898. 10.1021/es020839

Lakhani, A., 2012. Source apportionment of particle bound polycyclic aromatic hydrocarbons at an industrial location in Agra, India. Sci. World J. 1–10. http://dx.doi.org/10.1100/2012/781291

Lee, H.J., An, S., Kim, G.B., 2014. Background level and composition of polybrominated diphenyl ethers (PBDEs) in creek and subtidal sediments in a rural area of Korea. Sci. Total Environ. 470, 1479–1484. https://doi.org/10.1016/j.scitotenv.2013.06.105

Li, F., Zeng, X., Yang, J., Zhou, K., Zan, Q., Lei, A., Tam, N.F.Y., 2014. Contamination of polycyclic aromatic hydrocarbons (PAHs) in surface sediments and plants of mangrove swamps in Shenzhen, China. Mar. Pollut. Bull. 85, 590–596. https://doi.org/10.1016/j.marpolbul.2014.02.025

Liou, M.L., Yeh, S.C., Ling, Y.C., Chen, C.M., 2006. The Need for Strategic Environmental Assessment of Fishery Products Regulations in the Taiwan Strait: Taking Health Perspectives of Organochlorine Pesticides in Seafood as an Example, Human and Ecological Risk Assessment: An International Journal. 12(2), 390-401. 10.1080/10807030500536827

Lipiatou, E., Saliot, A., 1991. Fluxes and transport of anthropogenic and natural polycyclic aromatic hydrocarbons in the western Mediterranean Sea. Mar Chem. 32(1), 51–71. 10.1016/0304-4203(91)90025-r

Liu, D., Wu, S., Xu, H., Zhang, Q., Zhang, S., Shi, L., Yao, C., Liu, Y., Cheng, J., 2017. Distribution and bioaccumulation of endocrine disrupting chemicals in water, sediment and fishes in a shallow Chinese freshwater lake: Implications for ecological and human health risks. Ecotoxicol. Environ. Saf. 140, 222–229. https://doi.org/10.1016/j.ecoenv.2017.02.045

Long, E. R., Macdonald, D. D., Smith, S. L., Calder, F. D., 1995. Incidence of adverse biological effects within ranges of chemical concentrations in marine and estuarine sediments. Environ Manage, 19(1), 81–97.10.1007/bf02472006

Magi, E., Bianco, R., Ianni, C., Di Carro, M., 2002. Distribution of polycyclic aromatic hydrocarbons in the sediments of the Adriatic Sea. Environ. Pollut. 119(1), 91–98. 10.1016/s0269-7491(01)00321-9

Mai, B.X., Chen, S.J., Luo, X.J., Chen, L.G., Yang, Q.S., Sheng, G.Y., Peng, P.G., Fu, J., Zeng, E.Y., 2005. Distribution of polybrominated diphenyl ethers in sediments of the Pearl River Delta and adjacent South China Sea, Environ. Sci. Technol. 39, 3521–3527. 10.1021/es048083x

Maioli, O.L.G., Rodrigues, K.C., Knoppers, B.A., Azevedo, D.A., 2010. Polycyclic aromatic and aliphatic hydrocarbons in *Mytella charruana*, a bivalve mollusk from Mundaú lagoon, Brazil. Microchem. J. 96, 172–179. https://doi.org/10.1016/j.microc.2010.03.001

Martí, M., Ortiz, X., Gasser, M., Martí, R., Montaña, M.J., Díaz-Ferrero, J., 2010. Persistent organic pollutants (PCDD/Fs, dioxin-like PCBs, marker PCBs, and PBDEs) in health supplements on the Spanish market. Chemosphere 78(10), 1256–1262. https://doi.org/10.1016/j.chemosphere.2009.12.038

McVeety, B. D., Hites, R. A., 1988. Atmospheric deposition of polycyclic aromatic hydrocarbons to water surfaces: A mass balance approach. Atmos. Environ. 22(3), 511–536. 10.1016/0004-6981(88)90196-5

Miglioranza, K. S. ., de Moreno, J. E. ., Moreno, V. J., 2004. Organochlorine pesticides sequestered in the aquatic macrophyte *Schoenoplectus californicus* (C.A. Meyer) Soják from a shallow lake in Argentina. Water Res. 38(7), 1765–1772. 10.1016/j.watres.2004.01.017

Minh, N.H., Isobe, T., Ueno, D., Matsumoto, K., Mine, M., Kajiwara, N., Takahashi, S., Tanabe, S., 2007. Spatial distribution and vertical profile of polybrominated diphenyl ethers and hexabromocyclododecanes in sediment core from Tokyo Bay, Japan. Environ. Pollut. 148, 409–417. https://doi.org/10.1016/j.envpol.2006.12.011

Moon, H.-B., Kannan, K., Lee, S.-J., Choi, M., 2007. Polybrominated diphenyl ethers (PBDEs) in sediment and bivalves from Korean coastal waters. Chemosphere 66, 243–251. https://doi.org/10.1016/j.chemosphere.2006.05.025

Nakata, H., Sakai, Y., Miyawaki, T., Takemura, A., 2003. Bioaccumulation and Toxic Potencies of Polychlorinated Biphenyls and Polycyclic Aromatic Hydrocarbons in Tidal Flat and Coastal Ecosystems of the Ariake Sea, Japan. Environ. Sci. Technol. 37(16), 3513 3521. 10.1021/es021083h

Nascimento, R.A., Almeida, M., Escobar, N.C.F., Ferreira, S.L.C., Mortatti, J., Queiroz, A.F.S., 2017. Sources and distribution of polycyclic aromatic hydrocarbons (PAHs) and organic matter in surface sediments of an estuary under petroleum activity influence, Todos os Santos Bay, Brazil. Mar. Pollut. Bull. 119, 223–230. https://doi.org/10.1016/j.marpolbul.2017.03.069

Nelson, C., Drouillard, K., Cheng, K., Elliott, J., Ismail, N., 2015. Accumulation of PBDEs in an urban river otter population and an unusual finding of BDE-209. Chemosphere. 118, 322–328. 10.1016/j.chemosphere.2014.10.007

Nguyen, T.C., Loganathan, P., Nguyen, T.V., Vigneswaran, S., Kandasamy, J., Stevenson, D.S.G., Naidu, R., 2014. Polycyclic aromatic hydrocarbons in road-deposited sediments, water sediments, and soils in Sydney, Australia: comparisons of concentration distribution, sources and potential toxicity. Ecotoxicol. Environ. Saf. 104, 339–348. https://doi.org/10.1016/j.ecoenv.2014.03.010

Ohta, S., Nakao, T., Nishimura, H., Okumura, T., Aozasa, O., Miyata, H., 2002. Contamination levels of PBDEs, TBBPA, PCDDs/DFs, PBDDs/DFs and PXDDs/DFs in the environment of Japan. Organohalogen Compd. 57, 57–60.

Oros, D.R., Hoover, D., Rodigari, F., Crane, D., Sericano, J., 2005. Levels and distribution of polybrominated diphenyl ethers in water, surface sediments, and bivalves from the San Francisco Estuary. Environ. Sci. Technol. 39, 33–41. 10.1021/es048905q

Pan, J., Yang, Y.L., Xu, Q., Chen, D.Z., Xi, D.L., 2007. PCBs, PCNs and PBDEs in sediments and mussels from Qingdao coastal sea in the frame of current circulations and influence of sewage sludge. Chemosphere 66, 1971–1982. https://doi.org/10.1016/j.chemosphere.2006.07.070

Parkerton, T. F.; Connolly, J. P.; Thoman, R. V.; Uchin, C. G., 1993. Do aquatic effects or human health end points govern the development of sediment-quality criteria for nonionic organic chemicals? Environ. Toxicol. Chem. 12, 507-523. https://doi.org/10.1002/etc.5620120312

Pereira, W.E., Hostettler, F.D., Rapp, J.B., 1996. Distribution and fate of chlorinated insecticides, biomarkers and polycyclic aromatic hydrocarbons in sediments along a contamination gradient from a point source in San Francisco Bay, California. Mar Environ Res. 41, 299–314.

Peruzzo, P. J., Porta, A. A., Ronco, A. E., 2008. Levels of glyphosate in surface waters, sediments and soils associated with direct sowing soybean cultivation in north pampasic region of Argentina. Environ. Pollut. 156(1), 61–66. 10.1016/j.envpol.2008.01.015

Pikkarainen, A.L., 2007. Polychlorinated biphenyls and organochlorine pesticides in Baltic Sea sediments and bivalves. Chemosphere 68, 17–24. https://doi.org/10.1016/j.chemosphere.2006.12.079

Ponce-Velez, G., Botello, A.V., Diaz-Gonzalez G., 2006. Organic and inorganic pollutants in marine sediments from northern and southern continental shelf of the Gulf of Mexico. Int J Environ Pollut. 26 (1-3), 295. 10.1504/IJEP.2006.009113.

Pozo, K., Oyola, G., Estellano, V. H., Harner, T., Rudolph, A., Prybilova, P., Focardi, S., 2017. Persistent Organic Pollutants (POPs) in the atmosphere of three Chilean cities using passive air samplers. Sci Total Environ. 586, 107–114. 10.1016/j.scitotenv.2016.11.054

Rajendran, R.B., Imagawa, T., Tao, H., Ramesh, R., 2005. Distribution of PCBs, HCHs and DDTs, and their ecotoxicological implications in Bay of Bengal, India. Environ. Int. 31, 503–512. https://doi.org/10.1016/j.envint.2004.10.009

Ramdine, G., Fichet, D., Louis, M., Lemoine, S., 2012. Polycyclic aromatic hydrocarbons (PAHs) in surface sediment and oysters (*Crassostrea rhizophorae*) from mangrove of Guadeloupe: levels, bioavailability, and effects. Ecotoxicol. Environ. Saf. 79, 80–89. https://doi.org/10.1016/j.ecoenv.2011.12.005

Ramzi, A., Habeeb, R.K., Gireeshkumar, T.R., Balachandran, K.K., Jacob Chacko

Chandramohanakumar, N., 2017. Dynamics of polycyclic aromatic hydrocarbons (PAHs) in surface sediments of Cochin estuary, India. Mar. Pollut. Bull. 114, 1081–1087. https://doi.org/10.1016/j.marpolbul.2016.10.015

Salgueiro-González, N., Turnes-Carou, I., Besada, V., Muniategui-Lorenzo, S., López- Mahía, P., Prada-Rodríguez, D., 2015. Occurrence, distribution and bioaccumulation of endocrine disrupting compounds in water, sediment and biota samples from a European river basin. Sci. Total Environ. 529, 121–130. https://doi.org/10.1016/j.scitotenv.2015.05.048

Sánchez-Avila, J., Vicente, J., Echavarri-Erasun, B., Porte, C., Tauler, R., Lacorte, S., 2013. Sources, fluxes and risk of organic micropollutants to the Cantabrian Sea (Spain). Mar. Pollut. Bull. 72, 119–132. https://doi.org/10.1016/j.marpolbul.2013.04.010

Sarkar, A., Nagarajan, R., Chaphadkar, S., Pal, S., Singbal, S.Y.S., 1997. Contamination of organochlorine pesticides in sediments from the Arabian Sea along the west coast of India. Water Res. 31, 195–200. https://doi.org/10.1016/S0043-1354(96)00210-2

Sbriz, L., Aquino, M. R., Alberto de rodriguez, N. M., Fowler, S. W., Sericano, J. L., 1998. Levels of chlorinated hydrocarbons and trace metals in bivalves and nearshore sediments from the Dominican Republic. Mar. Pollut. Bull. 36(12), 971–979. 10.1016/s0025-326x(98)00097-6

Senthilkumar, K., Kannan, K., Subramanian, A., Tanabe, S., 2001. Accumulation of organochlorine pesticides and polychlorinated biphenyls in sediments, aquatic organisms, birds, bird eggs and bat collected from South India. Environ Sci Pollut Res Int. 8(1), 35 47. 10.1007/bf02987293

Sifakis, S., Androutsopoulos, V.P., Tsatsakis, A.M., Spandidos, D.A., 2017. Human exposure to endocrine disrupting chemicals: effects on the male and female reproductive systems. Environ. Toxicol. Pharmacol. 51, 56–70. https://doi.org/10.1016/j.etap.2017.02.024

Simcik, M. F., Eisenreich, S. J., Lioy, P. J., 1999. Source apportionment and source/sink relationships of PAHs in the coastal atmosphere of Chicago and Lake Michigan. Atmos. Environ. 33(30), 5071–5079.10.1016/s1352-2310(99)00233-2

Song, W., Ford, J.C., Li, A., Mills, W.J., Buckley, D.R., Rockne, K.J., 2004. Polybrominated diphenyl ethers in the sediments of the great lakes, Lake Superior. Environ. Sci. Technol. 38, 3286–3293.

Song, W., Ford, J.C., Li, A., Sturchio, N.C., Rockne, K.J., Buckley, D.R., Mills, W.J., 2005. Polybrominated diphenyl ethers in the sediments of the great lakes. 3. Lakes Ontario and Erie. Environ. Sci. Technol. 39, 5600–5605. 10.1021/es050631z

Soukarieh, B., El Hawari, K., El Husseini, M., Budzinski, H., Jaber, F., 2018. Impact of Lebanese practices in industry, agriculture and urbanization on soil toxicity. Evaluation of the

Polycyclic Aromatic Hydrocarbons (PAHs) levels in soil. Chemosphere. 210, 85–92. 10.1016/j.chemosphere.2018.06.178

Tombesi, N., Pozo, K., Álvarez, M., Přibylová, P., Kukučka, P., Audy, O., Klánová, J., 2017. Tracking polychlorinated biphenyls (PCBs) and polybrominated diphenyl ethers (PBDEs) in sediments and soils from the southwest of Buenos Aires Province, Argentina (South eastern part of the GRULAC region). Sci Total Environ. 575, 1470–1476. 10.1016/j.scitotenv.2016.10.013

Tongo, I., Ezemonye, L., Akpeh, K., 2017. Levels, distribution and characterization of polycyclic aromatic hydrocarbons (PAHs) in Ovia River, southern Nigeria. J. Environ. Chem. Eng. 5, 504–512. https://doi.org/10.1016/j.jece.2016.12.035

Van Ael, E., Covaci, A., Blust, R., Bervoets, L., 2012. Persistent organic pollutants in the Scheldt estuary: Environmental distribution and bioaccumulation. Environ Int. 48, 17–27.10.1016/j.envint.2012.06.017

Verweij, F., Booij, K., Satumalay, K., van der Molen, N., van der Oost, R., 2004. Assessment of bioavailable PAH, PCB and OCP concentrations in water, using semipermeable membrane devices (SPMDs), sediments and caged carp. Chemosphere. 54, 1675–1689. https://doi.org/10.1016/j.chemosphere.2003.10.002

Voorspoels, S., Covaci, A., Maervoet, J., De Meester, I., Schepens, P., 2004. Levels and profiles of PCBs and OCPs in marine benthic species from the Belgian North Sea and the Western Scheldt Estuary. Mar. Pollut. Bull. 49(5-6), 393–404. 10.1016/j.marpolbul.2004.02.024

Wang, X.T., Chen, L., Wang, X.K., Zhang, Y., Zhou, J., Xu, S.Y., Sun, Y.F., Wu, M.H., 2015. Occurrence, profiles, and ecological risks of polybrominated diphenyl ethers (PBDEs) in river sediments of shanghai, China. Chemosphere. 133, 22–30. https://doi.org/10.1016/j.chemosphere.2015.02.064

Whitall, D., Mason, A., Pait, A., Brune, L., Fulton, M., Wirth, E., Vandiver, L., 2014. Organic and metal contamination in marine surface sediments of guanica bay, Puerto Rico. Mar. Pollut. Bull. 80, 293–301. https://doi.org/10.1016/j.marpolbul.2013.12.053

Witt G., 1995. Polycyclic aromatic hydrocarbons in water and sediment of the Baltic Sea. Mar Pollut Bull. 31: 237–48. https://doi.org/10.1016/0025-326X(95)00174-L

Wright, J., Williams, S., Dethier, M., 2004. No zone is always greener: Variation in the performance of *Fucus gardneri* embryos, juveniles and adults across tidal zone and season. Mar Biol. 145, 1061–1073. https://doi.org/10.1007/s00227-004-1399-2

World Health Organisation handbook, 1979; WHO Offset, publication No. 48.

Wurl, O., Obbard, J.P., 2005. Organochlorine pesticides, polychlorinated biphenyls and polybrominated diphenyl ethers in Singapore's coastal marine sediments. Chemosphere 58, 925–933. https://doi.org/10.1016/j.chemosphere.2004.09.054

Yang, H., Xue, B., Jin, L., Zhou, S., Liu, W., 2011. Polychlorinated biphenyls in surface sediments of Yueqing Bay, Xiangshan Bay, and Sanmen Bay in East China Sea. Chemosphere 83, 137–143. https://doi.org/10.1016/j.chemosphere.2010.12.070

Yeh H.C., Chen, I.M., Chen, P., Wang W.H., 2009. Heavy metal concentrations of the soldier crab (*Mictyris brevidactylus*) along the inshore area of Changhua, Taiwan. Environ Monit Assess. 153, 103–109. 10.1007/s10661-008-0340-4

Yuan, D., Yang, D., Wade, T. L., Qian, Y., 2001. Status of persistent organic pollutants in the sediment from several estuaries in China. Environ. Pollut. 114(1), 101–111. 10.1016/s0269-7491(00)00200-1

Yuan, T.H., Shie, R.H., Chin, Y.Y., Chan, C.C., 2015. Assessment of the levels of urinary 1-hydroxypyrene and air polycyclic aromatic hydrocarbon in PM2.5 for adult exposure to the petrochemical complex emissions. Environ. Res. 136, 219-226. https://doi.org/10.1016/j.envres.2014.10.007

Yuan, H., Liu, E., Zhang, E., Luo, W., Chen, L., Wang, C., Lin, Q., 2017. Historical records and sources of polycyclic aromatic hydrocarbons (PAHs) and organochlorine pesticides (OCPs) in sediment from a representative plateau lake, China. Chemosphere 173, 78–88. https://doi.org/10.1016/j.chemosphere.2017.01.047

Yunker, M., Macdonald, R., Goyette, D., Paton, D., Fowler, B., Sullivan, D., Boyd, J., 1999. Natural and anthropogenic inputs of hydrocarbons to the Strait of Georgia. Sci Total Environ. 225(3), 181–209. 10.1016/s0048-9697(98)00362-3

Yunker, M. B., Macdonald, R. W., Vingarzan, R., Mitchell, R. H., Goyette, D., Sylvestre, S., 2002. PAHs in the Fraser River basin: a critical appraisal of PAH ratios as indicators of PAH source and composition. Org. Geochem. 33(4), 489–515.10.1016/s0146-6380(02)00002-5

Zanaroli, G., Negroni, A., Häggblom, M. M., Fava, F., 2015. Microbial dehalogenation of organohalides in marine and estuarine environments. Curr. Opin. Biotechnol. 33, 287–295. 10.1016/j.copbio.2015.03.013

Zhao, L., Hou, H., Zhou, Y., Xue, N., Li, H., Li, F., 2010. Distribution and ecological risk of polychlorinated biphenyls and organochlorine pesticides in surficial sediments from Haihe River and Haihe Estuary Area, China. Chemosphere 78, 1285–1293. https://doi.org/10.1016/j.chemosphere.2009.12.007

Zhao, G., Zhou, H., Liu, X., Li, K., Zhang, P., Wen, W., Yu, Y., 2012. PHAHs in 14 principal river sediments from Hai River basin, China. Sci. Total Environ. 427, 139–145. https://doi.org/10.1016/j.scitotenv.2012.04.001

Zheng, B., Liu, R., Liu, Y., Jin, F., An, L., 2015. Phenolic endocrine-disrupting chemicals and intersex in wild crucian carp from Hun River, China. Chemosphere 120, 743–749. https://doi.org/10.1016/j.chemosphere.2014.10.049

Zhou, J.L., Hong, H., Zhang, Z., Maskaoui, K., Chen, W., 2000. Multiphase distribution of organic micropollutants in Xiamen Harbour, China. Water Res. 34, 2132–2150. https://doi.org/10.1016/S0043-1354(99)00360-7.

Figure

Click here to download Figure fig1.jpg

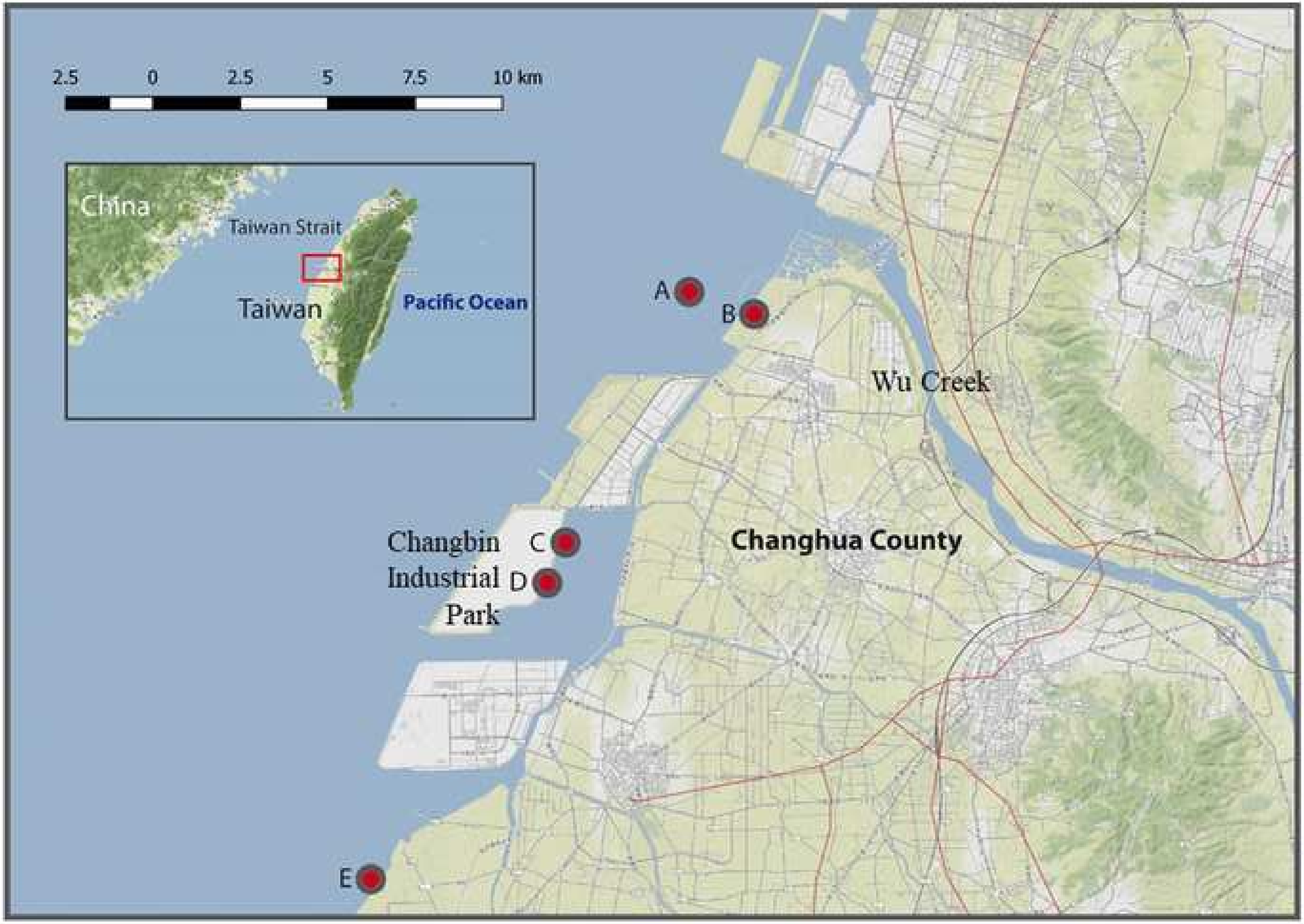

Fig.1 Sampling stations (A, B, C, D, and E) at the western coast of central Taiwan.

Figure

Click here to download Figure fig2.GIF

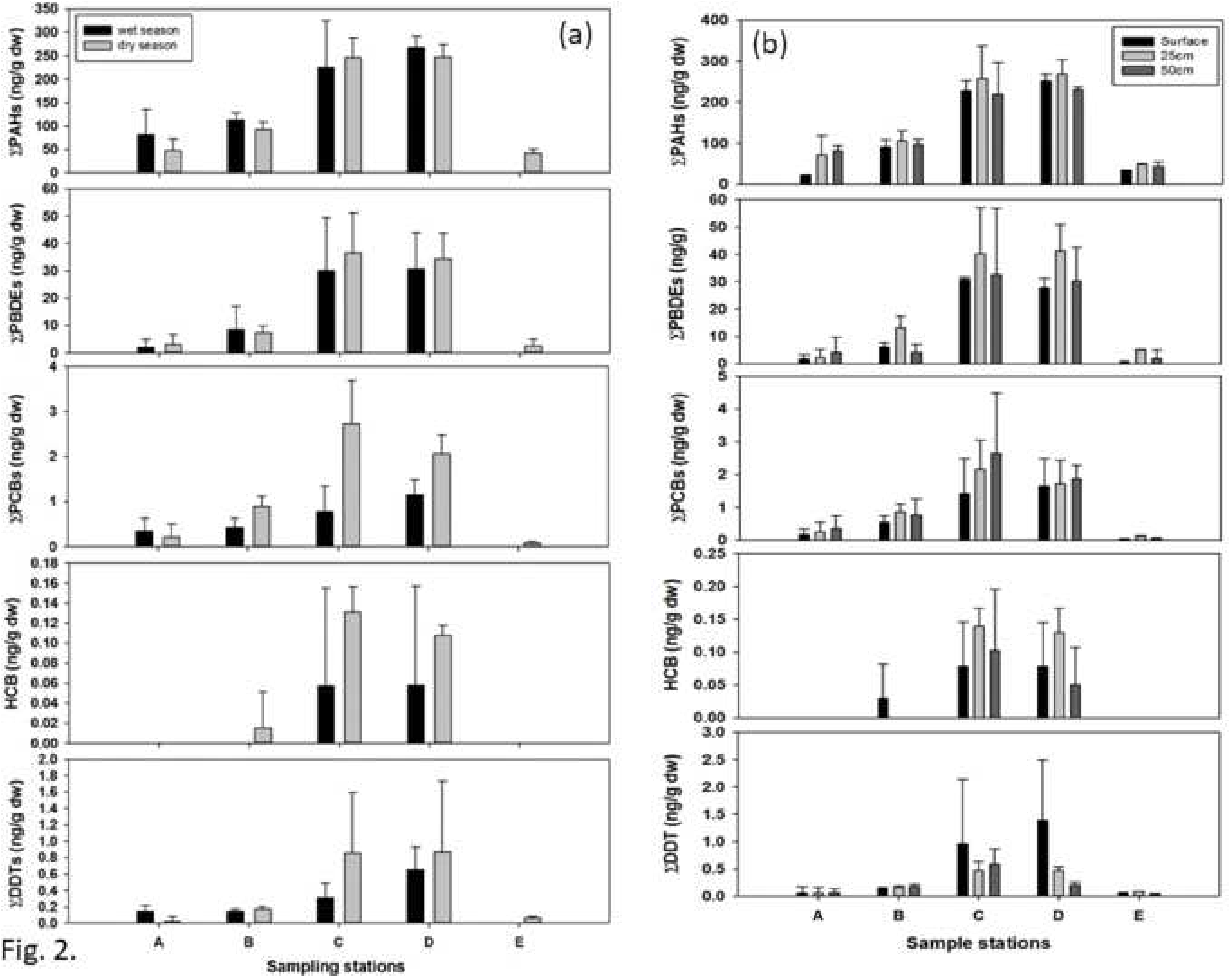

Fig. 2.

 Click here to download Figure fig3.GIF

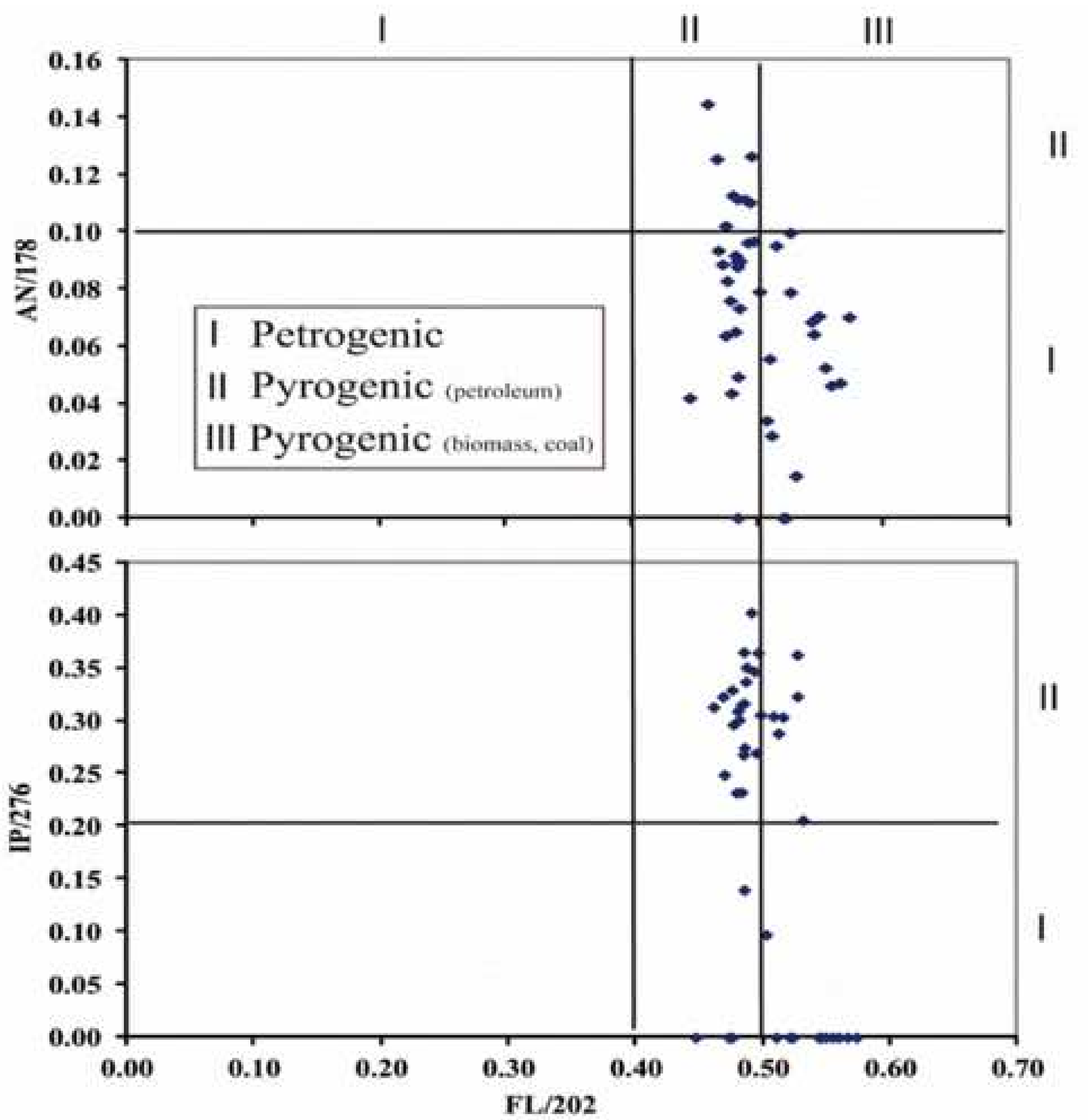

Fig. 3.

Figure2

Click here to download Figure fig4.GIF

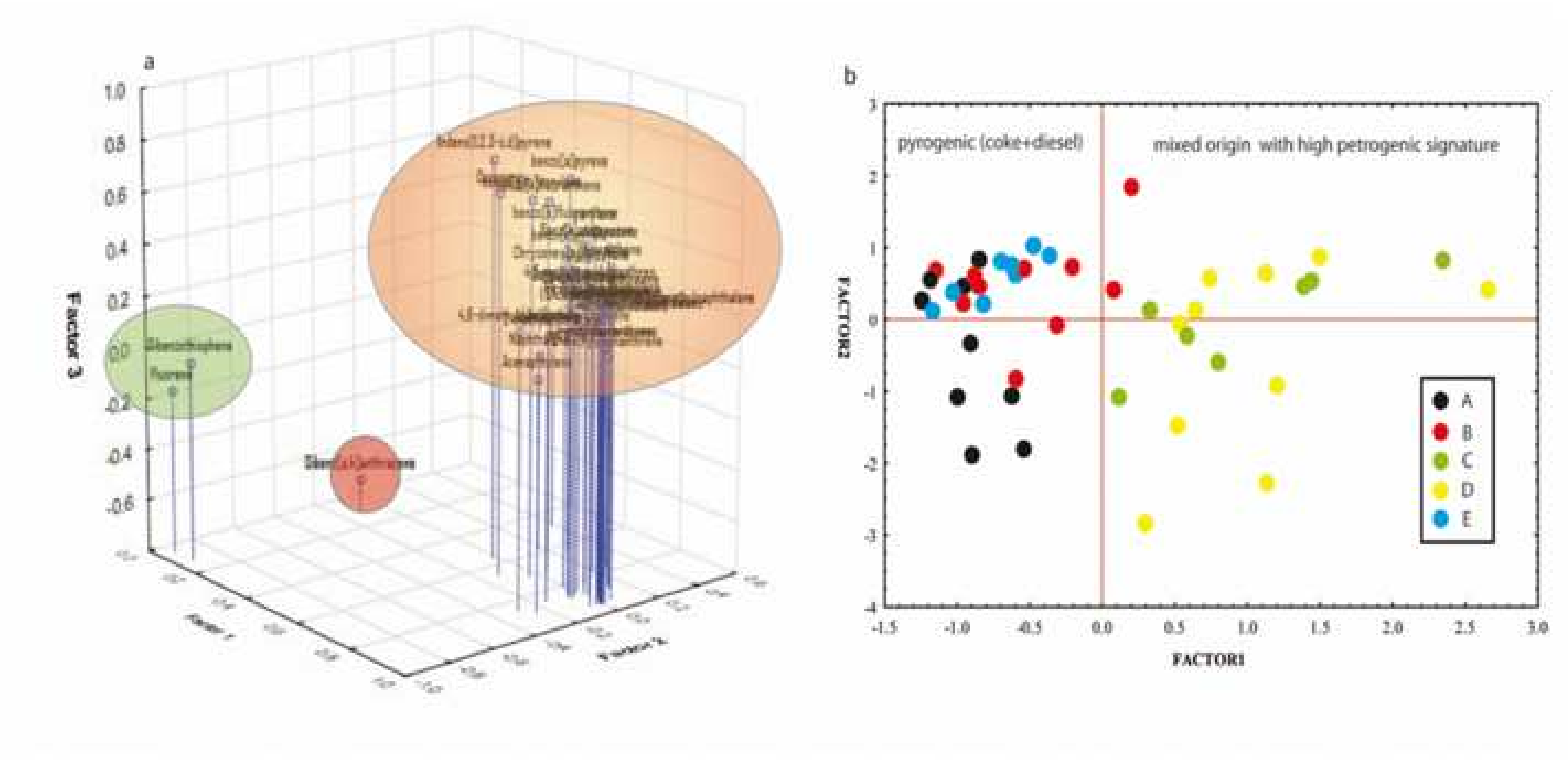

Fig. 4.

Figure

Click here to download Figure fig5.GIF

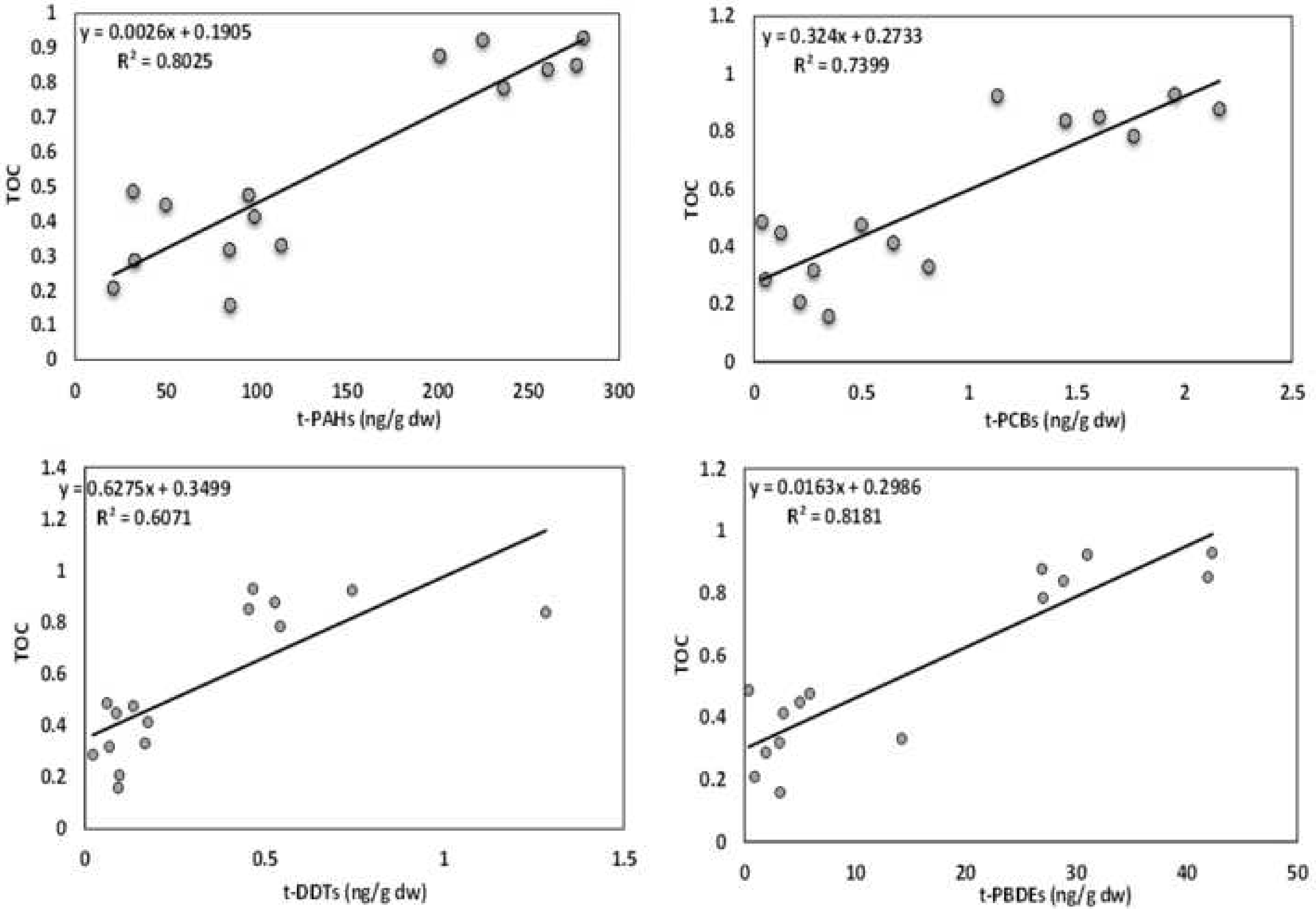

Fig. 5.

Click here to access/download
Supporting Information
Supplementary data.docx
