# Supplementary material for: Occurrence and distribution of anthropogenic persistent organic pollutants in coastal sediments and mud shrimps from the wetland of central Taiwan
Source: PLoS One. 2020 Jan 9;15(1):e0227367. doi: 10.1371/journal.pone.0227367 (PMC6956766; doi:10.1371/journal.pone.0227367)
Supplement: Attachment — Submitted filename: response to reviewers.docx [file pone.0227367.s008.docx]

**Letter from Editor:**

PONE-D-19-27465
Occurrence and distribution of anthropogenic persistent organic pollutants in coastal sediments and mud shrimps from the wetland of central Taiwan
PLOS ONE
Dear Dr. Ko,
Thank you for submitting your manuscript to PLOS ONE. After careful consideration, we feel that it has merit but does not fully meet PLOS ONE’s publication criteria as it currently stands. Therefore, we invite you to submit a revised version of the manuscript that addresses the points raised during the review process.
We would appreciate receiving your revised manuscript by Dec 02 2019 11:59PM. When you are ready to submit your revision, log on to https://www.editorialmanager.com/pone/ and select the 'Submissions Needing Revision' folder to locate your manuscript file.
If you would like to make changes to your financial disclosure, please include your updated statement in your cover letter.
To enhance the reproducibility of your results, we recommend that if applicable you deposit your laboratory protocols in protocols.io, where a protocol can be assigned its own identifier (DOI) such that it can be cited independently in the future. For instructions see: <http://journals.plos.org/plosone/s/submission-guidelines#loc-laboratory-protocols>
Please include the following items when submitting your revised manuscript:

- A rebuttal letter that responds to each point raised by the academic editor and reviewer(s). This letter should be uploaded as separate file and labeled 'Response to Reviewers'.
- A marked-up copy of your manuscript that highlights changes made to the original version. This file should be uploaded as separate file and labeled 'Revised Manuscript with Track Changes'.
- An unmarked version of your revised paper without tracked changes. This file should be uploaded as separate file and labeled 'Manuscript'.

Please note while forming your response, if your article is accepted, you may have the opportunity to make the peer review history publicly available. The record will include editor decision letters (with reviews) and your responses to reviewer comments. If eligible, we will contact you to opt in or out.

We look forward to receiving your revised manuscript.
Kind regards,
Hans-Uwe Dahms, Ph.D.
Academic Editor
PLOS ONE

Response: Thanks for the reminder. The following is the detailed response to the journal requirements and the reviewers’ concerns and suggestions.

Journal Requirements:

When submitting your revision, we need you to address these additional requirements.

1. Please ensure that your manuscript meets PLOS ONE's style requirements, including those for file naming. The PLOS ONE style templates can be found at http://www.journals.plos.org/plosone/s/file?id=wjVg/PLOSOne_formatting_sample_main_body.pdf and <http://www.journals.plos.org/plosone/s/file?id=ba62/PLOSOne_formatting_sample_title_authors_affiliations.pdf>

Response: Yes, the revision has been adjusted to meet the standards of PLOS ONE’s requirements.

2. We noticed you have some minor occurrence of overlapping text with the following previous publication(s), which needs to be addressed: https://link.springer.com/article/10.1007%2Fs10661-008-0696-5, https://link.springer.com/article/10.1007%2Fs11356-018-04113-x. In your revision ensure you cite all your sources (including your own works), and quote or rephrase any duplicated text outside the methods section. Further consideration is dependent on these concerns being addressed.

Response: Yes, thanks for the comments. The references have been cited, and the duplicated text have been rephrased in the revision.

3. Please amend your Methods section to clarify how the mud shrimp were collected, stored, transported and sacrificed.

Response: Collection and treatment of mud shrimp samples in methods section are updated with the cited references.

4. Please amend your Financial disclosure statement to declare sources of funding, or state that the authors received no specific funding.

Response: The financial disclosure statement has been added to declare sources of funding.

5. In your Methods section, please provide additional location information, including geographic coordinates for the data set if available.

Response: Yes, the information of sampling cites can be found in the supplementary data in Table S1.

6. In your Methods section, please provide additional information regarding the permits you obtained for the work. Please ensure you have included the full name of the authority that approved the field site access and, if no permits were required, a brief statement explaining why.

Response: The permits for sampling mud shrimps is not required. The information is added in the revision text (Line 101-102).

7.  We note that Figure 1in your submission contain [map/satellite] images which may be copyrighted. All PLOS content is published under the Creative Commons Attribution License (CC BY 4.0), which means that the manuscript, images, and Supporting Information files will be freely available online, and any third party is permitted to access, download, copy, distribute, and use these materials in any way, even commercially, with proper attribution. For these reasons, we cannot publish previously copyrighted maps or satellite images created using proprietary data, such as Google software (Google Maps, Street View, and Earth). For more information, see our copyright guidelines: http://journals.plos.org/plosone/s/licenses-and-copyright.

We require you to either (1) present written permission from the copyright holder to publish these figures specifically under the CC BY 4.0 license, or (2) remove the figures from your submission:

1.    You may seek permission from the original copyright holder of Figure 1 to publish the content specifically under the CC BY 4.0 license.

We recommend that you contact the original copyright holder with the Content Permission Form (http://journals.plos.org/plosone/s/file?id=7c09/content-permission-form.pdf) and the following text:

“I request permission for the open-access journal PLOS ONE to publish XXX under the Creative Commons Attribution License (CCAL) CC BY 4.0 (http://creativecommons.org/licenses/by/4.0/). Please be aware that this license allows unrestricted use and distribution, even commercially, by third parties. Please reply and provide explicit written permission to publish XXX under a CC BY license and complete the attached form.”

Please upload the completed Content Permission Form or other proof of granted permissions as an "Other" file with your submission.

In the figure caption of the copyrighted figure, please include the following text: “Reprinted from [ref] under a CC BY license, with permission from [name of publisher], original copyright [original copyright year].”

2.    If you are unable to obtain permission from the original copyright holder to publish these figures under the CC BY 4.0 license or if the copyright holder’s requirements are incompatible with the CC BY 4.0 license, please either i) remove the figure or ii) supply a replacement figure that complies with the CC BY 4.0 license. Please check copyright information on all replacement figures and update the figure caption with source information. If applicable, please specify in the figure caption text when a figure is similar but not identical to the original image and is therefore for illustrative purposes only.

The following resources for replacing copyrighted map figures may be helpful:

USGS National Map Viewer (public domain): http://viewer.nationalmap.gov/viewer/

The Gateway to Astronaut Photography of Earth (public domain): http://eol.jsc.nasa.gov/sseop/clickmap/

Maps at the CIA (public domain): https://www.cia.gov/library/publications/the-world-factbook/index.html and https://www.cia.gov/library/publications/cia-maps-publications/index.html

NASA Earth Observatory (public domain): http://earthobservatory.nasa.gov/

Landsat: http://landsat.visibleearth.nasa.gov/

USGS EROS (Earth Resources Observatory and Science (EROS) Center) (public domain): http://eros.usgs.gov/#

Natural Earth (public domain): <http://www.naturalearthdata.com/>

Response: The image in figure 1 was generated from DIVA-GIS, using free spatial data and QGIS version 2.6, and is not copyrighted.

8. Please include captions for your Supporting Information files at the end of your manuscript, and update any in-text citations to match accordingly. Please see our Supporting Information guidelines for more information: <http://journals.plos.org/plosone/s/supporting-information>.

Response: Thanks for your comments. The captions for the supporting information files have been added at the end of the manuscript (line 662-663).

Additional Editor Comments (if provided):
The MS entitled "Occurrence and distribution of anthropogenic persistent organic pollutants in coastal sediments and mud shrimps from the wetland of central Taiwan" submitted to the journal PLoSone investigates the source and distribution of various organic pollutants in mud shrimps and sediments around the industrial area, west coast of central Taiwan. They concluded that concentrations of pollutants were greater in samples collected near the industrial area than those from the non-industrial locations and also revealed recent DDT inputs to the area. Considering the aim, precise methodology and outcomes, the MS may be accepted for publication with minor revisions.

The authors should consider the comments given by the reviewers below and return a REVISED VERSION with an accompanying AUTHOR RESPONSE files until the 1st December 2019.
HANS-U. DAHMS, 17th October 2019
Response: Thanks so much for the comments. The manuscript has been revised and resubmitted to PLos One for publication.

Reviewers' comments:
Reviewer's Responses to Questions
**Comments to the Author**
1. Is the manuscript technically sound, and do the data support the conclusions?
The manuscript must describe a technically sound piece of scientific research with data that supports the conclusions. Experiments must have been conducted rigorously, with appropriate controls, replication, and sample sizes. The conclusions must be drawn appropriately based on the data presented.
Reviewer #1: Yes
Reviewer #2: Yes
Reviewer #3: Yes

Response: Thanks.

2. Has the statistical analysis been performed appropriately and rigorously?
Reviewer #1: Yes
Reviewer #2: Yes
Reviewer #3: Yes

Response: Thanks.

3. Have the authors made all data underlying the findings in their manuscript fully available?
The [PLOS Data policy](http://www.plosone.org/static/policies.action#sharing) requires authors to make all data underlying the findings described in their manuscript fully available without restriction, with rare exception (please refer to the Data Availability Statement in the manuscript PDF file). The data should be provided as part of the manuscript or its supporting information, or deposited to a public repository. For example, in addition to summary statistics, the data points behind means, medians and variance measures should be available. If there are restrictions on publicly sharing data—e.g. participant privacy or use of data from a third party—those must be specified.
Reviewer #1: Yes
Reviewer #2: Yes
Reviewer #3: Yes

Response: Thanks.

4. Is the manuscript presented in an intelligible fashion and written in standard English?
PLOS ONE does not copyedit accepted manuscripts, so the language in submitted articles must be clear, correct, and unambiguous. Any typographical or grammatical errors should be corrected at revision, so please note any specific errors here.
Reviewer #1: Yes
Reviewer #2: Yes
Reviewer #3: Yes

Response: Thanks.

5. Review Comments to the Author
Please use the space provided to explain your answers to the questions above. You may also include additional comments for the author, including concerns about dual publication, research ethics, or publication ethics. (Please upload your review as an attachment if it exceeds 20,000 characters)

Reviewer #1: The MS entitled "Occurrence and distribution of anthropogenic persistent organic pollutants in coastal sediments and mud shrimps from the wetland of central Taiwan" submitted to the journal PLoSone investigates the source and distribution of various organic pollutants in mud shrimps and sediments around the industrial area, west coast of central Taiwan. They concluded that concentrations of pollutants were greater in samples collected near the industrial area than those from the non-industrial locations and also revealed recent DDT inputs to the area. Considering the aim, precise methodology and outcomes, the MS may be accepted for publication with minor revisions.

Response: Thanks for your suggestions. The minor revisions of this manuscript had been completed.

Specific comments:
Use degree symbols instead of using superscript of zero (e.g. line no. 97-98)
In this study mud shrimps were also collected to check the pollutants. There might be water and/or sediment particles adhered on the body of the shrimps which may have trace amount of pollutants. Before homogenizing them, how authors removed the adhered water and/or sediment particles? Pls mention clearly in the methods section (line 103)

Response: The degree symbols have been corrected (lines 92, 93, and 100). The description has been rewritten in method section (lines 97-102).

Study area map (Figure 1) looks convincing; however authors should add the position of the map and lat long details in the figure

Response: The location and characteristics of sampling cites including latitudes and longitudes are described in the supplementary data at Table S1.

Line 168-170: The sentence “Figure 2a shows the concentration of different POPs....... season (January)” may be transferred to end of the paragraph, after line no. 178

Response: Thanks for the suggestion. This sentence has been moved to the end of this paragraph (lines 180-182).

Line 172 & Tables: Pls define what is dw (dry weight?) in the MS

Response: Yes, the dw has been defined as dry weight at the first time appear in the text (line 173).

Line 218: change “and analyze relationships” to “and to analyze relationships”

Response: Thanks for the suggestion. It has been approved (line 219).

Line 219: change “PC1 accounted for the major proportion” to “PC1 accounted major proportion”

Response: Thanks for the suggestion. It has been approved (line 220).

Line 251: change “may originate from” to “may be originated from”

Response: Thanks for the suggestion. It has been approved (line 247).

Line 254: add a reference to the sentence “These results are consistent with our previous findings”

Response: The description has been rewritten.

Line 361: the formula mentioned here may be moved to the methods section

Response: Thanks for the suggestions. The formula has been moved to the methods section.

Reviewer #2: This manuscript presented a novel and distinctive study on the POPs in coastal sediments and mud shrimps from the wetland of central Taiwan with supporting results. The paper is suitable for publication with following corrections:
1. Add some of the most important quantitative results to the Abstract.

Response: Thanks for the suggestion. We previously included the bioaccumulation factors for DDTs and PCBs. We have added in quantitative results for PAH concentrations in heavily contaminated site compared to less contaminated sites.

2. The discussion section should cite all the recent references.

Response: Thanks for the suggestions. The discussion section has been rewritten.

3. Authors should carefully review the final resolution of the figures prior to publication for better understanding of results. Especially Fig.4 is not clear.

Response: Thanks for the comments. Figure 4 has been corrected to 2D, instead of 3D providing a clear resolutions.

4. Cite the source of future investigations in this study.

Response: Thanks for the suggestion. Citations have been reviewed and updated.

5. The authors are suggested to make changes according to the comments appended in the manuscript’s PDF format.

Response: Thanks for the suggestions. The revision has been improved according your valuable suggestions.

6. Rectify grammatical errors to reach up to international standards.

Response: The manuscript has been carefully polished by correcting the grammatical errors.

7. Ensure that the references and whole manuscript is as per journal format. Remove hyperlink from references.

Response: The reference format has been adjusted to match the publishing standard of PLos One.

Reviewer #3: This manuscript reported the concentrations of several POPs in sediment and mud shrimps in a wetland of west coast of central Taiwan, China. It could provide useful information for POPs distribution and risk assessment in wetlands. Generally, it is well organized and written.

However, the manuscript should be revised before acceptance for publication. Details are as follows.
(1) The language should be improved. "At" should be used rather than "in" before "station" or "site".

Response: Thanks for the suggestions. The preposition “in” has been replaced by “at”, before sampling stations or sites as suggestion.

(2) "POPs" should be used over the whole text instead of "POP".

Response: Thanks for the comments. It has been adjusted to consistently use “POPs”.

(3) Why chose the five stations? The authors should state their sampling design more clearly. Besides, the ambient environment of the study area should be described.

Response: The characteristics of the sampling sites are described in the Table S1 in the supplementary data.

(4) Page 15-16, the method for BAFs calculation should be moved to the Material and methods part.

Response: Thanks for the suggestions. The BAF equation has been moved to the Materials and methods section.

(5) It's OK to combine results and discussion together. However, the discussion is very surficial. The authors should make deeper discussion, especially for the mechanism of the distribution pattern of POPs.

Response: Results and discussion section has been rewritten and updated.

(6) For Table 1, sediment type should be mentioned in each study.

Response: The sediment information in Table 1 are from the coastal or estuarine areas.

(7) For Fig. 1, the longitude and latitude should be added on the map. The resolution should be improved.

Response: Yes, the Figure 1 have been updated with longitude and latitude.

(8) Fig. 4 is difficult to read. Please improve the quality.

Response: Yes, the quality of Figure 4 has been improved.

6. PLOS authors have the option to publish the peer review history of their article ([what does this mean?](https://journals.plos.org/plosone/s/editorial-and-peer-review-process#loc-peer-review-history)). If published, this will include your full peer review and any attached files.
If you choose “no”, your identity will remain anonymous but your review may still be made public.
**Do you want your identity to be public for this peer review?** For information about this choice, including consent withdrawal, please see our [Privacy Policy](https://www.plos.org/privacy-policy).
Reviewer #1: No
Reviewer #2: No
Reviewer #3: Yes: Xiaoshou Liu
[NOTE: If reviewer comments were submitted as an attachment file, they will be attached to this email and accessible via the submission site. Please log into your account, locate the manuscript record, and check for the action link "View Attachments". If this link does not appear, there are no attachment files to be viewed.]

While revising your submission, please upload your figure files to the Preflight Analysis and Conversion Engine (PACE) digital diagnostic tool, <https://pacev2.apexcovantage.com/>. PACE helps ensure that figures meet PLOS requirements. To use PACE, you must first register as a user. Registration is free. Then, login and navigate to the UPLOAD tab, where you will find detailed instructions on how to use the tool. If you encounter any issues or have any questions when using PACE, please email us at [figures@plos.org](mailto:figures@plos.org). Please note that Supporting Information files do not need this step.
