# Supplementary material for: Occurrence and distribution of anthropogenic persistent organic pollutants in coastal sediments and mud shrimps from the wetland of central Taiwan
Source: PLoS One. 2020 Jan 9;15(1):e0227367. doi: 10.1371/journal.pone.0227367 (PMC6956766; doi:10.1371/journal.pone.0227367)
Supplement: Attachment — Submitted filename: plos reviewer comments 14.10.19.docx [file pone.0227367.s007.docx]

This manuscript presented a novel and distinctive study on the POPs in coastal sediments and mud shrimps from the wetland of central Taiwan with supporting results. The paper is suitable for publication with following corrections:  

1. Add some of the most important quantitative results to the Abstract.
2. The discussion section should cite all the recent references.
3. Authors should carefully review the final resolution of the figures prior to publication for better understanding of results. Especially Fig.4 is not clear.
4. Cite the source of future investigations in this study.
5. The authors are suggested to make changes according to the comments appended in the manuscript’s PDF format.
6. Rectify grammatical errors to reach up to international standards.
7. Ensure that the references and whole manuscript is as per journal format. Remove hyperlink from references.
